# Optimized glycemic control of type 2 diabetes with reinforcement learning: a proof-of-concept trial

**DOI:** 10.1038/s41591-023-02552-9

**Published:** 2023-09-14

**Authors:** Guangyu Wang, Xiaohong Liu, Zhen Ying, Guoxing Yang, Zhiwei Chen, Zhiwen Liu, Min Zhang, Hongmei Yan, Yuxing Lu, Yuanxu Gao, Kanmin Xue, Xiaoying Li, Ying Chen

**Affiliations:** 1 Ministry of Education Key Laboratory of Metabolism and Molecular Medicine, Department of Endocrinology and Metabolism, Zhongshan Hospital, Fudan University, Shanghai, China; 2 State Key Laboratory of Networking and Switching Technology, Beijing University of Posts and Telecommunications, Beijing, China; 3 Big Data and Artificial Intelligence Center, Zhongshan Hospital, Fudan University, Shanghai, China; 4 Department of Endocrinology, XuHui Central Hospital of Shanghai, Shanghai, China; 5 Department of Endocrinology and Metabolism, Qingpu Branch of Zhongshan Hospital affiliated to Fudan University, Shanghai, China; 6 Big Data and Biomedical AI Laboratory, College of Future Technology, Peking University, Beijing, China; 7 Nuffield Department of Clinical Neurosciences, University of Oxford, Oxford, UK; 8 Shanghai Key Laboratory of Metabolic Remodeling and Health, Institute of Metabolism and Integrative Biology, Fudan University, Shanghai, China

**Keywords:** Therapeutics, Computational biology and bioinformatics

## Abstract

The personalized titration and optimization of insulin regimens for treatment of type 2 diabetes (T2D) are resource-demanding healthcare tasks. Here we propose a model-based reinforcement learning (RL) framework (called RL-DITR), which learns the optimal insulin regimen by analyzing glycemic state rewards through patient model interactions. When evaluated during the development phase for managing hospitalized patients with T2D, RL-DITR achieved superior insulin titration optimization (mean absolute error (MAE) of 1.10 ± 0.03 U) compared to other deep learning models and standard clinical methods. We performed a stepwise clinical validation of the artificial intelligence system from simulation to deployment, demonstrating better performance in glycemic control in inpatients compared to junior and intermediate-level physicians through quantitative (MAE of 1.18 ± 0.09 U) and qualitative metrics from a blinded review. Additionally, we conducted a single-arm, patient-blinded, proof-of-concept feasibility trial in 16 patients with T2D. The primary outcome was difference in mean daily capillary blood glucose during the trial, which decreased from 11.1 (±3.6) to 8.6 (±2.4) mmol L^−1^ (*P* < 0.01), meeting the pre-specified endpoint. No episodes of severe hypoglycemia or hyperglycemia with ketosis occurred. These preliminary results warrant further investigation in larger, more diverse clinical studies. ClinicalTrials.gov registration: NCT05409391.

## Abstract (web summary)

A reinforcement learning algorithm achieved superior insulin titration optimization versus other deep learning models and standard clinical methods in a prospective cohort and in a proof-of-concept feasibility trial.

## Main

Type 2 diabetes (T2D) is one of the most prevalent chronic diseases and leads to a considerable rate of death and social burden worldwide^1^. Patients with T2D with poor glycemic control require insulin therapy in the course of disease progression. Although good glycemic control can markedly reduce diabetic complications and mortality in hospitalized diabetic patients, it remains challenging and time-consuming to adjust insulin dosages within effective and safe limits^2,3^.

Although a series of clinical guidelines on rational insulin use for patients with T2D have been proposed by experts^4^, insulin dosage titration is typically based on the clinical guidance combined with physicians’ experience to achieve targeted glycemic goals^5^ and cannot fully take into consideration the variability for each patient in the real world^6,7^. Some treatment regimens may suit some patients better than others or only for some period of time for an individual as their disease condition progresses. Therefore, personalized and dynamic titration of insulin is of great clinical importance to reduce blood glucose fluctuations and prevent associated comorbidities and mortality in patients with T2D.

Artificial intelligence (AI) approaches have emerged as potentially powerful tools to aid in disease diagnosis and management^8–10^. Existing approaches have used supervised learning (SL), in which a list of correct labels must be provided, for disease detection or incidence prediction^11,12^. However, SL-based methods assume the expert performance to be optimal, which is not always consistent with real-world outcomes due to the complexity of human metabolism and differential responses to drugs among individuals.

Reinforcement learning (RL) has been proposed as a subfield of machine learning, enabling an agent to learn effective strategies through trial-and-error interactions with a dynamic environment^13^. RL could potentially offer an attractive solution for constructing adaptable policies in various healthcare domains, especially in the dynamic treatment regimens (DTRs) for long-term patient care^14^. With the increasing availability of medical record data, RL has been used in sequential medical decision-making systems in various clinical scenarios, including sepsis^15^, coronary heart disease^16^ and glycemic regulation by artificial pancreas systems^17^. Although several studies have used model-free RL models for treatment recommendation^18–20^, these approaches generally face challenges, such as sample efficiency and potential for unsafe policies when accurate simulation of the environment is lacking^21,22^. As safety is a primary concern in complex or long-term treatment scenarios, model-based RL might offer potential in simulating diverse scenarios, thereby providing reliable forward planning at decision time^22^. Despite RL methods’ potential in optimizing treatment regimen based on the reward set by patient outcomes, their real-world application in therapy remains limited due to potential risks in clinical practice^23,24^. Therefore, the incorporation of RL-based methods from development to adoption into the real-world clinical workflow requires comprehensive evaluation^25^.

**Fig. 1:**
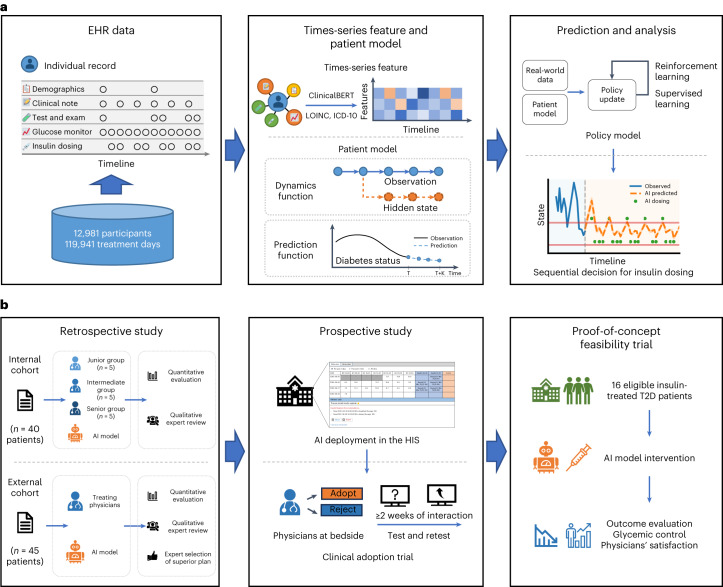
Schematic illustration of the AI system from development to deployment for dynamic insulin dosage titration for patients with T2D. **a**, Model development of the AI system—a model-based RL-DITR system consisting of ‘patient model’ and ‘policy model’. Left, we constructed a large multi-center EHR dataset consisting of records of long-term continuous clinical observation and medication of hospitalized patients with T2D. Middle, with the standardized time-series data as input, the patient model generated hidden state transition, status prediction and reward estimation. Right, the policy model is optimized by interacting with the patient model as an environment. **b**, Comprehensive evaluation of the AI system step-by-step for integration into the real-world clinical workflow. Left, we conducted multi-center retrospective studies, including quantitative and qualitative evaluations in the internal and external cohorts. Middle, a prospective study with test–retest was conducted in an academic hospital after AI deployment in the HIS. Right, a proof-of-concept feasibility trial was conducted to evaluate the glycemic control of and physician satisfaction with the AI system.

In the present study, we constructed a large dataset of electronic health records (EHRs) of hospitalized patients with T2D with continuous recording of insulin use protocols and glycemic response for at least 7 d. Every patient was represented as a temporal sequence of feature vector, including demographics, blood biochemical measurements, medications and insulin usage information. Based on the sequential inpatient EHR data, we developed an RL-based dynamic insulin titration regimen (RL-DITR), which consisted of a patient model to track an individual’s evolving glucose states and a policy model for multi-step planning in long-term care. This model-based RL approach learns the optimal policy by iteratively interacting with the patient model as the environment. Furthermore, we introduced SL to guarantee the safe states by using clinician expertise while optimizing outcomes through trial-and-error interactions with a dynamic environment, which could mimic and potentially augment the physicians in clinical decision-making. To evaluate our proposed AI system in clinical use^25^, we conducted stepwise clinical evaluations of the AI system in inpatient management from development to deployment, including (1) an internal validation of AI versus physician using both quantitative metrics and qualitative evaluations; (2) an external validation of AI versus physician using qualitative clinical evaluations with test–retest; (3) a prospective deployment study with test–retest; and (4) a final proof-of-concept feasibility clinical trial (Fig. 1). The clinical evaluations indicated that the RL-DITR system could potentially offer benefits in improving glycemic control for inpatients with T2D through dynamic management of subcutaneous insulin injections. Further investigation in larger, multi-center clinical studies is warranted to demonstrate generalizability of the tool.

## Results

### Dataset characteristics and system overview

A total of 12,981 inpatients with T2D with 119,941 treatment days were included in the AI model development phase analysis. The mean age was 59.2 ± 14.5 years, and 42.6% were females. The demographics and clinical characteristics of patients are presented in Extended Data Table 1.

To represent the patient information into a dynamic evolution process, we processed the patient data into multi-dimensional temporal standardized features. We used a ClinicalBERT pre-trained model and natural language processing (NLP) pipeline to extract the clinically relevant sequential features from real-world data (Methods). All the features were discretized to seven timesteps to obtain multi-dimensional temporal features (Fig. 1).

**Extended Data Fig. 1:**
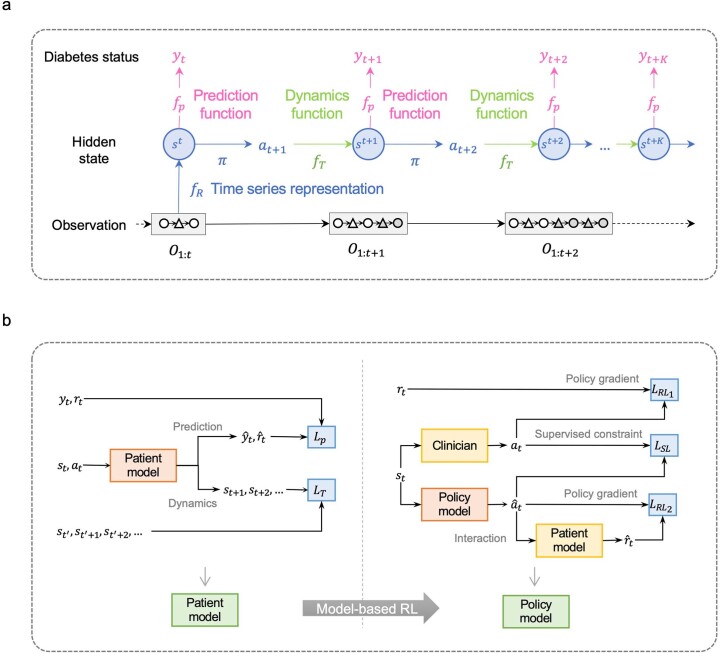
The development of the AI system. **a**, The sequential decision process with patient model and policy model in AI system. Given a trajectory, for the initial step, the representation function *f*_*R*_ receives as input the past observations *O*_1:*t*_ from the trajectory. The model is subsequently unrolled recurrently for K steps. At each step *κ*∈[1,*K*], the policy model π receives the hidden state ${s}_{t+k-1}$ and generates an action ${a}_{t+k-1}$. The dynamics function *f*_*T*_ of patient model subsequently receives as input the hidden state ${s}_{t+k-1}$ from the previous step and the action ${a}_{t+k-1}$ and produces the hidden state of the next step ${s}_{t+k}$, and the prediction function of patient model predicts diabetes status ${y}_{t+k-1}$. The hidden states and actions recurrently update. **b**, The AI system training pipeline. Left, the patient model learning for patient tracking. Given a hidden state *s*_*t*_ and an actual action ${a}_{t}$, the patient generates the predicted status ${\hat{y}}_{t}$ to estimate the current status *y*_*t*_, and produces the next state ${s}_{t+1}$. The estimated reward ${\hat{r}}_{t}$ compared to the actual reward *r*_*t*_ was calculated from ${\hat{y}}_{t}$. The patient model is joint optimized by the objective of consistency loss of state transition ${L}_{T}$ and supervised loss of status prediction ${L}_{p}$. Right, the policy update for dynamic regimen with combined supervised learning and reinforcement learning. The policy model *π* receives the hidden state ${s}_{t}$ and then generates an action ${\hat{a}}_{t}$. Subsequently, the patient model receives as input the hidden state$\,{s}_{t}$ from the previous step and the action *a*_*t*_, then produces the hidden state of the next step *s*_*t*+1_ and returns a reward ${\hat{r}}_{t}$. The policy model *π* is joint optimized by the objective of combined reinforcement learning ${L}_{R{L}_{1}}$, ${L}_{R{L}_{2}}$ and supervised learning *L*_*SL*_.

Our proposed RL-DITR as a model-based RL consists of two components: a patient model to characterize the diabetes progressive state by learning the environment’s dynamics and a policy model for management of diabetes by planning with respect to the learned model. Specifically, the patient model characterizes diabetes status via a dynamics function and a prediction function. Given the input of temporal features of a patient trajectory (for example, admission status, hospitalized observation and treatment plan) from admission to the timestep T, the dynamics function generated the hidden states of the patient. The hidden state is then updated iteratively and subsequently unrolled recurrently for ahead of K steps. At each timestep T + k (k < K), the prediction function receives as input the generated hidden state from the previous step and outputs the prediction of the clinical status, including blood glucose value and ‘within target range’ (WTR) for glycemia control (Fig. 1a and Extended Data Fig. 1). WTR indicates the blood glucose value within the target range 3.9–10.0 mmol L^−1^ for each timepoint^5,26^.

Then, we constructed the policy model to make multi-step planning for long-term care. At each step, the policy model is optimized by interacting with the patient model as an environment. The policy model was trained through a fusion of SL and patient model-based RL with joint learning. Through patient model-based RL, the policy model can learn individualized treatment trajectories and improve long-term clinical outcome. At the same time, it learns the treatment practices of clinicians in treating patients with T2D within a reasonable range of dosages by SL.

### Performance of AI model to predict patient glycemic states

**Fig. 2:**
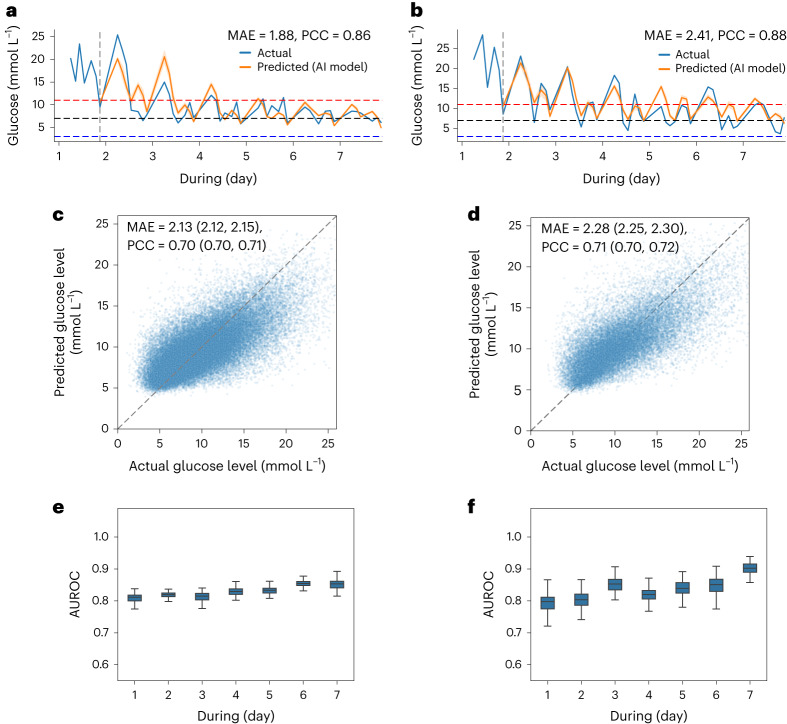
Performance of AI model system in the prediction of patient state trajectory. **a**,**b**, Comparison of actual patient trajectories and model-based state roll-outs for patients from the internal test set (**a**) and the external test set (**b**). Each predicted value, based on an individual patient, is generated within K steps from the last timestep of the previous day (K = 7 for 1 d ahead of time). The blue curve is measured patient glucose values, and the orange curve is predicted glucose values. **c**,**d**, Correlation analysis of the predicted glucose value versus the actual glucose value generated using the AI glucose model in the internal test set (**c**) and the external test set (**d**). **e**,**f**, ROC curves showing performance of daily WTR prediction on the internal test set (**e**) (*n* = 20,961 treatment days) and the external test set (**f**) (*n* = 16,077 treatment days). Each predicted value is based on the last timestep of the previous day. Box plots show the median (center lines), interquartile range (hinges) and 1.5× interquartile range (whiskers) (bootstrapping with *n* = 1,000 resamples). Each value generated by our RL-DITR system represents an individual-level prediction. These were then aggregated to produce population-level results. The correlation analysis is shown with 95% CIs in **c** and **d**. AUROC, area under the receiver operating characteristic; ROC, receiver operating characteristic.

To build a dynamic and individualized AI clinician for managing patients with T2D, we constructed the model-based RL framework. We first tested whether a patient’s glucose trajectories could be predicted by the patient model with two validation sets, including an internal test set and an external test set. For the comparison of actual state trajectories and model-based state roll-outs, the predicted glucose values follow the transition tendency accurately in both the internal test and the external test set (Fig. 2a,b). Case study results also suggest that the AI model was able to generate personalized information of a patient’s glucose state in terms of large timestep (k = 7). For the overall glucose prediction, we aggregated the individual-level prediction to produce population-level results, which were then used for further analysis. The AI model demonstrated good performance in the internal test set, achieving a Pearson correlation coefficient (PCC) of 0.70 (95% confidence interval (CI): 0.70, 0.71) and a mean absolute error (MAE) of 2.13 (95% CI: 2.12, 2.15) mmol L^−1^ (Fig. 2c). When evaluated on the external test set, the AI model achieved a PCC of 0.71 (95% CI: 0.70, 0.72) and an MAE of 2.28 (95% CI: 2.25, 2.30) mmol L^−1^ (Fig. 2d). As shown in Extended Data Table 2, the results indicate that our model outperformed the other baseline models with a substantial improvement.

**Extended Data Fig. 2:**
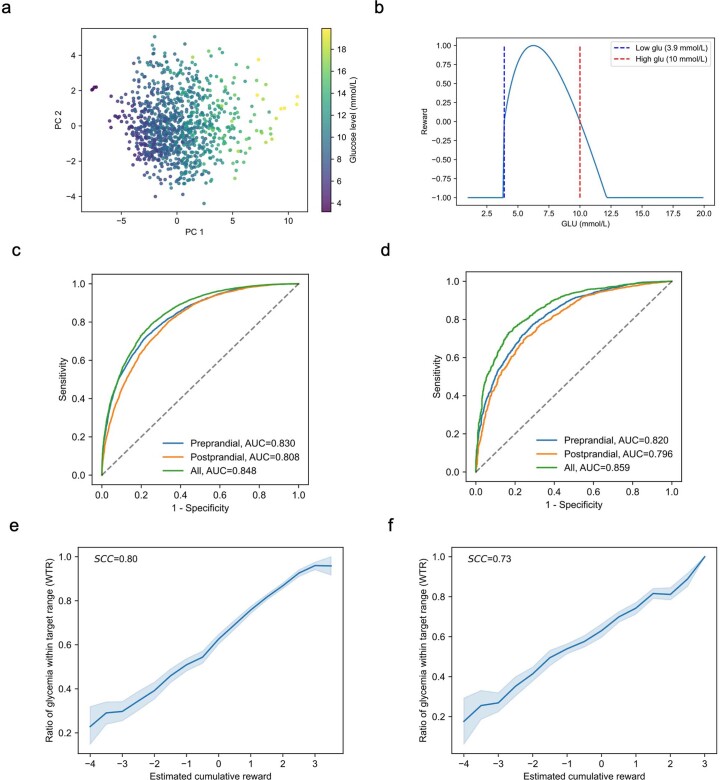
The performance evaluation of the AI system for patient trajectory prediction using WTR for overall glucose variability. **a**, Visualization of the patient hidden states. Projection of patients’ hidden state embeddings onto PC 1 and PC 2, derived from principal component analysis (PCA). Each node indicates a patient state. The state distribution showed association with diabetic outcome, colored by glucose level distribution. The samples are 1000 patients from internal test dataset. PC: principal component. **b**, Illustration of reward function. It is a measurement of overall glucose variability that focus on the relationship between glucose variability and risks for hypo- and hyperglycemia. **c** and **d**, Performance of the AI model on assessment of WTR shown as AUC curves. **c**, internal test set and **d**, external test set. ROC curves showing the pre-prandial time, the postprandial and overall performance. **e-f**, Correlation analysis of the ratio of glycemia within target range (WTR) vs the estimated cumulative reward of the clinicians’ treatment actions. **e**, internal test set, and **f**, external test set. The shaded area represents the 95% confidence interval.

We further validated the AI’s performance in predicting WTR (3.9–10.0 mmol L^−1^) of glucose values, which physicians can use to evaluate an individual’s overall insulin response and avoid the risk of hyperglycemic or hypoglycemic events. When evaluated using the internal test set, the AI model achieved an area under the curve of 0.830 for the prediction of WTR of preprandial blood glucose values, 0.808 for the prediction of WTR of postprandial blood glucose values and 0.848 for the average performance (Extended Data Fig. 2c,d). The model showed reliable performance validated on the external test set. We further investigated the model performance on predicting daily WTR status (glucose values within the target range of 3.9–10.0 mmol L^−1^ over the past 24 h) of patients along the timeline (Fig. 2e,f). We observed that the model becomes more accurate with more information input about a patient as time goes on.

We investigated the correlation between the patient outcome (WTR ratio) and the cumulative rewards estimated by the patient model. The AI model demonstrated good performance with a Spearman correlation coefficient (SCC) of 0.80 in the internal test set and an SCC of 0.73 in the external test set (Extended Data Fig. 2e,f). We observed that treatment actions with low cumulative rewards were associated with a low rate of WTR ratio, whereas treatments with high cumulative rewards achieved better glucose outcome with a high rate of WTR ratio. The results show that the patient model evaluation is highly correlated with the clinical outcome and could be used as the interaction environment for the RL model.

### Insulin titration optimization using model-based RL system

**Fig. 3:**
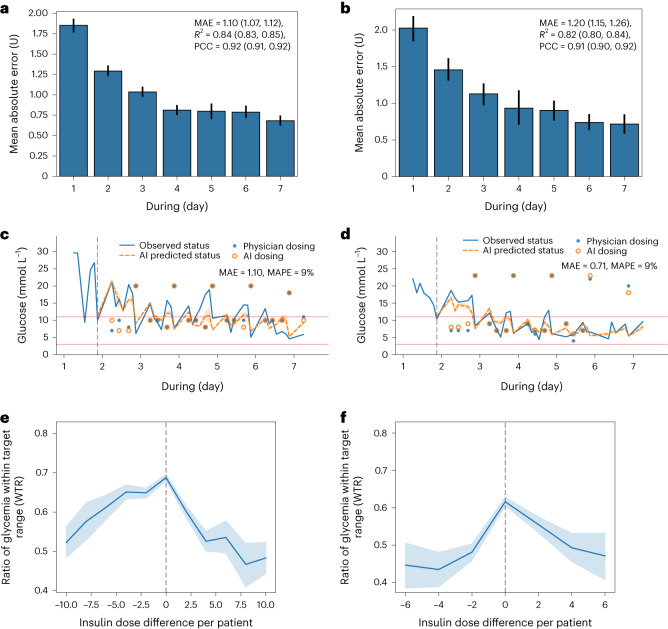
Performance of AI treatment model in the insulin dosage prediction. **a**,**b**, Performance of daily treatment dosage prediction on the internal test set (**a**) (*n* = 42,037 insulin data points) and the external test set (**b**) (*n* = 32,484 insulin data points). Each predicted value was subsequently unrolled recurrently for K steps from the last timestep of the previous day (K = 7 for 1 d ahead of time). The error bars represent the 95% CIs. We aggregated the individual-level predictions to obtain population-level results. **c**,**d**, Comparison of actual treatment regimens and model-based treatment roll-outs of two individual patients from the internal test set (**c**) and the external test set (**d**). The blue curve is measured patient glucose values, and the orange curve is predicted glucose values given by the AI model. **e**,**f**, Association analysis of the patient outcome (for example, WTR) versus the dosage difference in treatment actions between the AI policy and the clinician policy for the internal test set (**e**) and the external test set (**f**). The dose excess, referring to the difference between the given and the AI model, suggested dose summed over per day for all patients. The shaded area represents the 95% CI. *R*^2^, coefficient of determination. MAPE, mean absolute percentage error.

**Extended Data Fig. 3:**
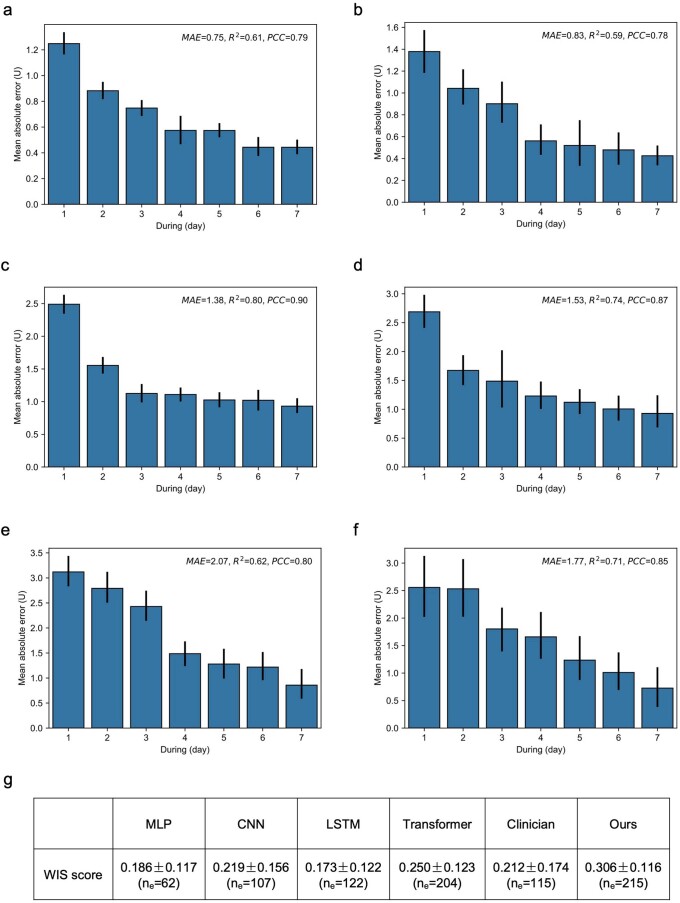
Performance of the AI for daily treatment dosage prediction and off-policy evaluation. **a-f**, Each column represents the performance of AI grouped by insulin types, including (**a** and **b**) short/rapid acting, (**c** and **d**) biphasic or premixed, and (**e** and **f**) long acting. Each predicted data is based on is generated within K steps based on the last time step of previous day (K = 7 for one day ahead of time). The bars represent the mean with 95% confidence intervals of MAE on the internal test set (*n* = 20,961 treatment days) and the external test set (*n* = 16,077 treatment days). MAE, mean absolute error; R^2^, coefficient of determination; PCC, Pearson’s correlation coefficient. **a, c** and **e**: the internal test set; **b, d** and **f**: external test set. **g**, Off-policy evaluation of RL-based model versus other SL-based and clinician methods in the internal test set in the AI development phase, measured by weighted importance sampling (WIS) score with standard deviation. ${n}_{e}$ indicates the effective sample size with the WIS score.

We further evaluated the RL system’s performance for optimization of personalized insulin recommendations. Figure 3a,b shows the correlation between the clinician policy and the AI policy in the development phase (internal and external test sets). For daily treatment dosage prediction, the AI policy achieved an MAE of 1.10 U (95% CI: 1.07, 1.12) on the internal test set and a good prediction performance on the external test set with an MAE of 1.20 U (95% CI: 1.15, 1.26). We found that the model becomes more accurate as the observed time window expands due to more trial-and-error interactions with the environment. The performance of the model was accurate when validated on different insulin types (including short/rapid-acting insulin, long-acting insulin and biphasic/premixed insulin) (Extended Data Fig. 3a–f).

Our proposed approach was then tested against several SL methods, including convolutional neural network (CNN), long-short term memory (LSTM), transformer and the standard clinical method. We found that our model-based RL method was able to export an accurate treatment regimen and outperformed other methods in the internal test set and the external test set (Extended Data Table 3). The results presented in Extended Data Table 3 demonstrate that our policy model, guided by our blood glucose model, outperformed other models substantially. We conducted further evaluation of the RL model by employing off-policy evaluation of weighted importance sampling (WIS)^27^, demonstrating the model’s superior performance in comparison to other methods (Extended Data Fig. 3g).

Figure 3c,d shows the dynamic treatment strategies generated by clinicians and model-based RL for two individual patients on different hospital days. The results demonstrated an overall trend of high similarity/correlation between the daily prescriptions of the clinicians and AI policies, indicating that AI was able to learn and mimic physician practice. We further investigated whether the patient outcome (WTR ratio) varied with the difference of the dose actually administered and the dose suggested by the RL method by correlation analysis (Fig. 3e,f). The results showed that patients who received doses similar to the doses recommended by the AI algorithm can typically achieve desired glucose control, both in the internal set (interquartile range, −2 to 0 U) and the external test set (interquartile range, 0–1 U). When the dose actually administered differed from the dose suggested by the AI algorithm, the average outcome got worse.

### Simulation study of performance of AI versus physicians

**Extended Data Fig. 4:**
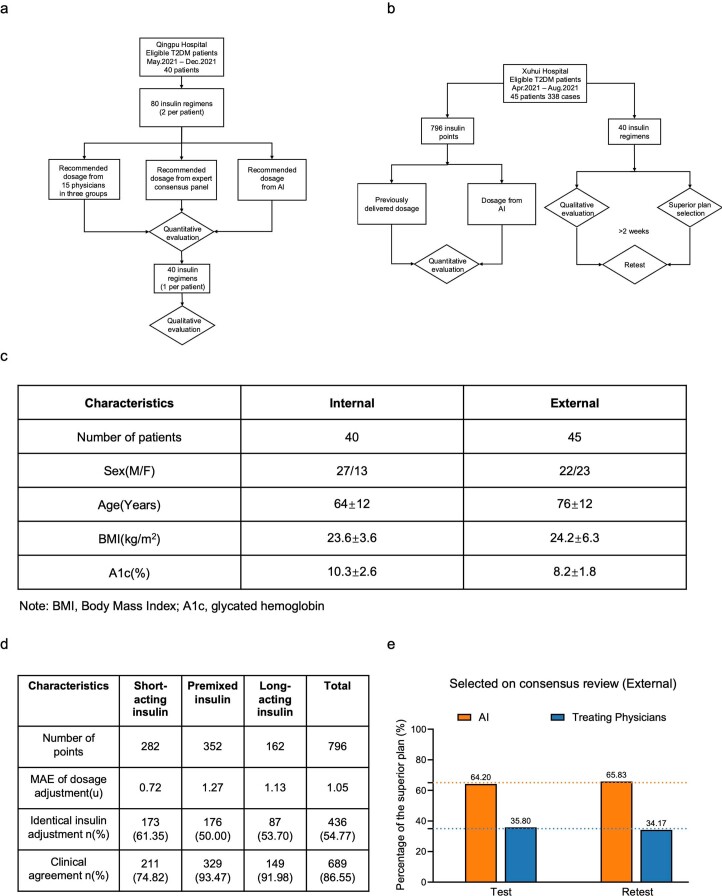
Performance evaluation of the AI system in the retrospective phase study of internal and external cohorts. **a**, Study design of internal cohort: 40 eligible T2D patients were included in the study. 80 treatment cases from 40 patients (2 per person) were randomly selected to compare the performance of quantitative metrics between the AI system and human physicians. 1 case for 40 patients was further selected for qualitative clinical evaluations (effectiveness, safety, and overall acceptability). **b**, Study design of external cohort: 45 T2D patients were collected, and a total of 796 insulin points were included in the external validation analysis. An assessment with quantitative metrics was conducted to compare the performance between treating physicians and AI by expert panel. 40 cases randomly selected from the total 338 cases were used for further qualitative evaluation (effectiveness, safety, and overall acceptability). After 2 weeks, a retest review was conducted. **c**, Demographics and baseline measurements of the patients in the internal (n = 40) and external (n = 45) cohorts. BMI, body mass index; A1c, glycated hemoglobin. Numerical variables were reported as mean±SD. **d**. Quantitative comparisons of insulin dosage given by human physicians and AI stratified by insulin catalogs in the external cohort. **e**, Superior plan **(**AI versus human physicians**)** was selected by the expert panel (n = 3) with test-retest review in the external cohort (n = 40 regimens). Orange dashed line, average performance of AI; blue dashed line, average performance of treating physicians. Bar graphs indicate the mean±SEM.

First, we conducted two retrospective simulation studies including an internal cohort and an external cohort for validation of the AI’s feasibility. For the internal validation cohort, we compared the performance between our AI system and human physicians in giving insulin dosage recommendation using 40 patients with T2D (with 226 insulin data points) (Extended Data Fig. 4a,c). A total of 15 physicians with different levels of clinical experience were enrolled and assigned to three groups: group 1, junior physicians with 1–3 years of clinical experience (*n* = 5); group 2, intermediate physicians with 4–7 years of clinical experience (*n* = 5); and group 3, senior physicians with 8–20 years of clinical experience (*n* = 5). RL-generated and physician-generated dosage titrations were evaluated by an expert panel, including quantitative metrics and qualitative metrics from clinical experience.

**Fig. 4:**
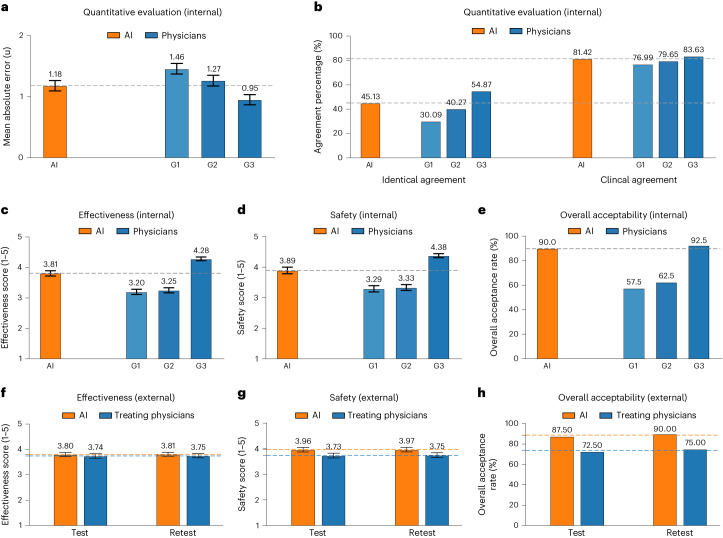
Performance evaluation between the AI model and human physicians in retrospective studies. **a**–**e**, Comparison with quantitative metrics (**a** and **b**) and qualitative clinical evaluations (**c**–**e**) on insulin titration regimens given by the AI model and human physician groups in the internal cohort (*n* = 40 patients with T2D). The AI model was compared with three physician groups with different levels of clinical experience: group 1, junior physicians (*n* = 5); group 2, intermediate physicians (*n* = 5); and group 3, senior physicians (*n* = 5). **a**,**b**, Quantitative evaluation of dosage titration by expert panel consensus as reference (*n* = 226 insulin data points). **a**, Predicted error (MAE) of AI model and human physicians; **b**, dosage adjustment agreement of the AI model and human physicians evaluated separately by identical agreement (same direction, same dosage) and clinical agreement (same direction, dose difference ≤20%). **c**–**e**, Qualitative clinical performances were evaluated by the expert panel (*n* = 40 regimens) separately in effectiveness (**c**), safety (**d**) and overall acceptability (**e**). **f**–**h**, Performance comparison of the AI-generated and previously delivered insulin regimens in the test–retest external cohort (*n* = 40 regimens). Evaluation was based on the expert panel review including effectiveness (**f**), safety (**g**) and overall acceptability (**h**). Orange dashed line represents the average performance of AI; blue dashed line represents the average performance of treating physicians. Bar graphs indicate the mean ± s.e.m. G, group.

Taking the dosage recommended by the expert panel as references, the MAE of the AI system was 1.18 U, outperforming junior physicians in group 1 with 1.46 U and intermediate physicians in group 2 with 1.27 U and slightly inferior to senior physicians in group 3 with 0.95 U (Fig. 4a). The percentage of ‘clinical agreement’ (defined as same direction, dose difference ≤20%) was 81.42% with the AI model, higher than that with group 1 (junior physicians) and slightly lower than that with group 3 (senior physicians). Likewise, the percentage of ‘identical agreement’ (defined as same direction, same dosage) with the AI system was higher than that with group 1 (junior physicians) (Fig. 4b).

The performance of RL and physicians’ treatment regimens was further evaluated by blinded consensus review using a subjective questionnaire, including effectiveness (if the regimen could control hyperglycemia, rated on Likert scale 1–5), safety (if the regimen could reduce hypoglycemia, rated on Likert scale 1–5) and overall acceptability (if the regimen would be acceptable for patient treatment). The perceived effectiveness, safety and acceptability of the AI model were higher than the junior and intermediate physician groups’ (group 1 and group 2) plans and slightly lower than those for the senior physician group’s (group 3) plans (Fig. 4c–e). These results suggest that our AI model is superior to junior physicians and similar to experienced physicians in the overall treatment regimen acceptability, hyperglycemia and hypoglycemia control.

Furthermore, we performed an external validation in 45 patients with T2D to compare the performance of AI plans and treating physician plans under a blinded review by an expert panel and by another blinded review for retesting at 2-week intervals at least (Extended Data Fig. 4b,c). The results demonstrated that the acceptability, effectiveness and safety of the AI plans were similar to the treating physicians who were board-certified endocrinologists, evaluated by subjective measurements made by an expert panel (Fig. 4f–h). Moreover, the AI system performed well across insulin categories (short/rapid-acting, biphasic/premixed and long-acting insulins) (Extended Data Fig. 4d). Then, a direct head-to-head comparison with test and retest review was conducted to select the superior plan (AI versus human physicians) by the expert panel review (*n* = 3) for the same cases. The percentage of selected superior AI plans was 64.2% in the test review and 65.8% in the retest review, suggesting that the AI model was superior to human plans based on expert evaluations (Extended Data Fig. 4e). These results demonstrate consistently superior performance of the AI model compared to its physician counterparts.

### Prospective deployment study of the AI system

**Fig. 5:**
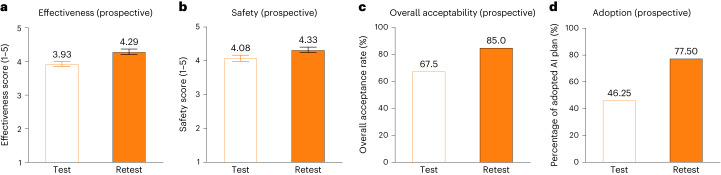
Performance evaluation of the AI model in the prospective study. **a**–**d**, The performance of effectiveness (**a**), safety (**b**), overall acceptability (**c**) and adoption rate of AI-generated regimen (**d**) evaluated by endocrinologists at the bedside at test–retest review in the prospective study (*n* = 40 regimens). The score scale of effectiveness and safety is 1–5. The adoption rate refers to the percentage of the AI regimens adopted by endocrinologists at the bedside for patient treatment. Bar graphs depict the mean ± s.e.m.

**Extended Data Fig. 5:**
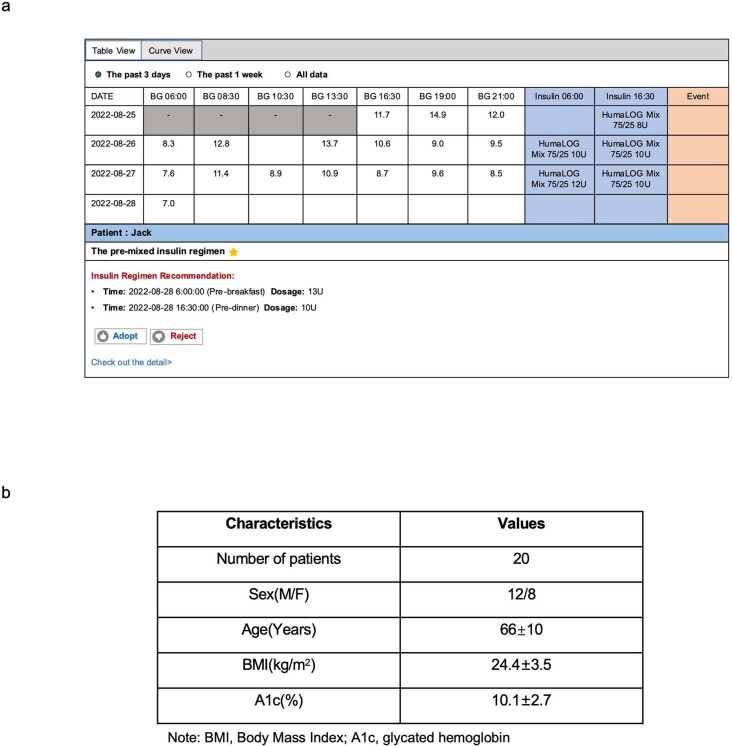
Performance evaluation of AI system in the prospective deployment study. **a**, The user interface of AI deployment. **b**, Demographics and baseline measurements of patients in the deployment phase study (n = 20). BMI, body mass index; A1c, glycated hemoglobin. Numerical variables were reported as mean±SD.

In the prospective deployment study in 20 patients with T2D, the AI system’s performance was evaluated by endocrinologists at the bedside, including effectiveness, safety and acceptability as well as the adoption rate. We used adoption rate to evaluate the percentage of the AI regimens adopted by endocrinologists for patient treatment. All the evaluations were under test–retest review with an interval of 2 weeks at least with human–machine interaction (Extended Data Fig. 5a,b). Our proposed RL model demonstrated stable performance of effectiveness, safety and acceptability over time, even better in the retest review (Fig. 5a–c).

Moreover, the AI regimen was deemed to be acceptable for about 70% of acceptance rate at the initial test review while the endocrinologists initially contacted with the AI system, which is similar to the acceptability in the simulation phases (Fig. 5c). Intriguingly, a higher adoption rate of 77.5% was attained at the retest review after a period of clinical practice with the AI system. Although the adoption rate of the AI plan was relatively low at the initial test review, we found an increase of 31.25% between the test–retesting intervals (Fig. 5d). These results suggested a step-by-step increase of trust of the AI treatment regimen by physicians through human–machine interaction, and the AI system was gradually adopted by physicians into routine clinical practice.

### Proof-of-concept feasibility clinical trial of the AI system

**Fig. 6:**
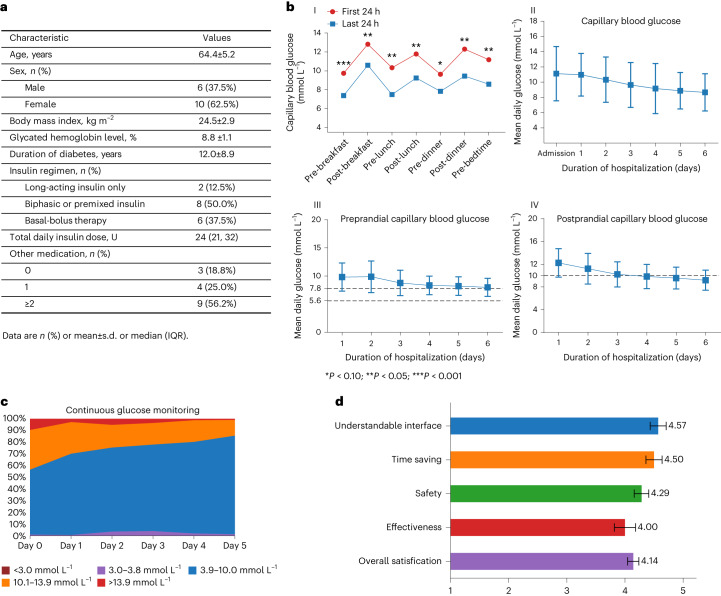
A proof-of-concept feasibility trial to evaluate the AI system on glycemic control in patients with T2D. **a**, The baseline clinical characteristics of patients with T2D included in the proof-of-concept feasibility trial (*n* = 16). **b**, The capillary blood glucose of a patient with T2D during the treatment period. (I) Illustration of the seven-point glucose profile during the first and last 24 h of the treatment period. Statistical significance was determined by two-sided paired *t*-test: pre-breakfast ***P < 0.001; post-breakfast, pre-lunch, post-lunch, post-dinner and pre-bedtime **P < 0.05; pre-dinner *P < 0.10. (II) Mean daily capillary blood glucose. (III) Mean preprandial capillary blood glucose. (IV) Mean postprandial capillary blood glucose during the treatment period. The preprandial blood glucose target was 5.6–7.8 mmol L^−1^; the postprandial capillary blood glucose target was <10.0 mmol L^−1^. (II–IV) Line, median; error bar, interquartile; *n* = 16 patients. **c**, Average percentage of continuous glucose monitoring data within glycemic ranges throughout the treatment period. The percentage of continuous glucose measurement <3.0 mmol L^−1^, 3.0–3.8 mmol L^−1^, 3.9–10.0 mmol L^−1^, 10.1–13.9 mmol L^−1^ and >13.9 mmol L^−1^ is presented. **d**, Post-intervention evaluation of the AI system during the treatment trial, assessed by physicians (*n* = 14) using questionnaires (see more in Extended Data Fig. 7c). The satisfaction agreement was scored from a scale of 1–5. Bar graphs indicate the mean ± s.e.m. IQR, interquartile range.

**Extended Data Fig. 6:**
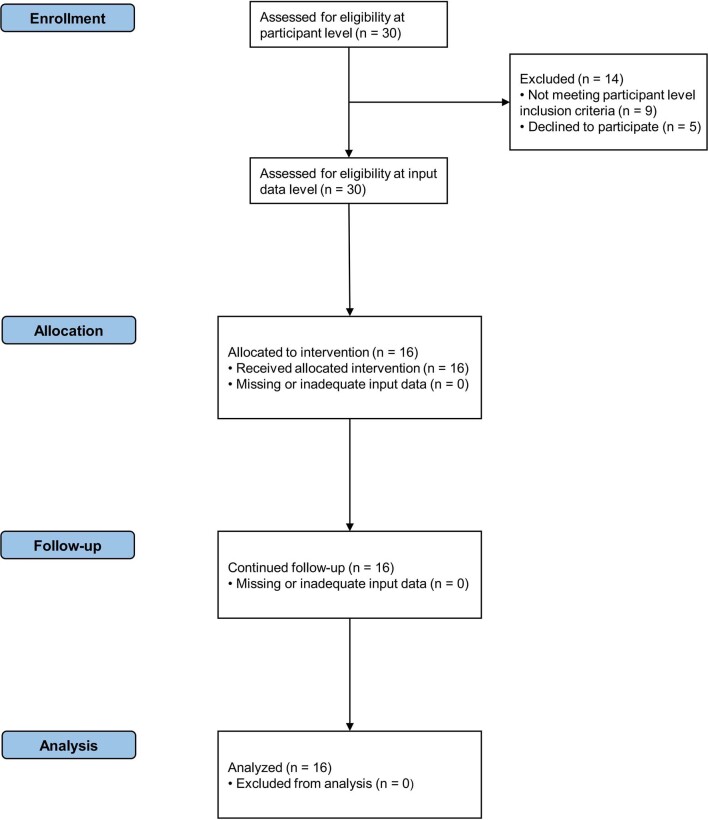
Flow diagram of the proof-of-concept feasibility trial.

A proof-of-concept feasibility trial was performed to investigate the clinical utility and safety of AI in hospitalized patients with T2D for glycemic control. Sixteen inpatients with T2D were enrolled in the trial (Extended Data Fig. 6). Their mean HbA1c was 8.8 ± 1.1% at baseline, and mean diabetes duration was 12.0 ± 8.9 years (Fig. 6a). All inpatients underwent 5 d of intervention by AI. Over the trial, 90.2% of the AI recommendations were adopted by physicians, indicating a high level of confidence in the algorithm’s dosing.

**Extended Data Fig. 7:**
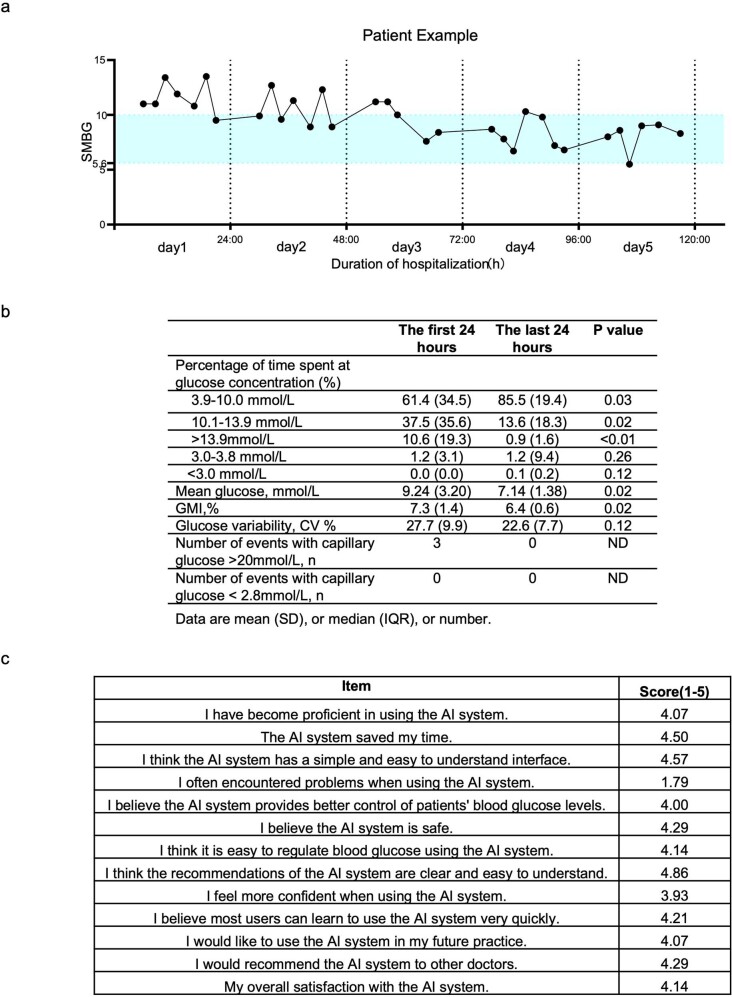
Performance evaluation of the AI system in the proof-of-concept feasibility trial. **a**, Patient example during the proof-of-concept feasibility trial using the seven-point capillary blood glucose measurement. **b**, Glucose control based on the sensor glucose measurements at the first 24 hours and the last 24 hours of the trial. GMI, glucose management indicator; CV, coefficient of variation. **c**, Post-intervention evaluation by physicians who used the AI during the feasibility trial. The post-intervention evaluation questionnaire included 13 items questions: 8 items for pertaining to the physician’s experience with the AI use and recommendation and 5 items for assessing the physician’s view regarding integration of the AI into daily routine practice.

A considerable improvement in seven-point capillary blood glucose profile was observed across timepoints in the last 24 h of the treatment period compared to the first 24 h of the treatment period (Fig. 6b). The mean daily capillary blood glucose, preprandial capillary blood glucose and postprandial capillary blood glucose were decreased with AI’s treatment, from the mean (±s.d.) of 11.1 (±3.6) mmol L^−1^ to 8.6 (± 2.4) mmol L^−1^, 10.2 (±2.8) mmol L^−1^ to 7.8 (±2.2) mmol L^−1^ and 12.3 (±4.2) mmol L^−1^ to 9.7 (±2.4) mmol L^−1^, respectively, in the first 24 h compared to the last 24 h of treatment (Fig. 6b), which achieved our pre-specified primary endpoint. At the end of the trial, 70.3% preprandial capillary blood glucose achieved the target range of 5.6–7.8 mmol L^−1^, and 68.8% postprandial capillary blood glucose achieved the target <10.0 mmol L^−1^. A patient example of the seven-point capillary blood glucose during the AI intervention is shown in Extended Data Fig. 7a.

We also used continuous glucose monitoring (CGM) for the evaluation of the algorithm-directed glycemic control for the secondary outcomes. The percentage of glucose concentration in time in range (TIR) (3.9–10.0 mmol L^−1^) by CGM was constantly improved, and the percentage of glucose concentration <3.9 mmol L^−1^ was less than 4% over the trial (Fig. 6c). TIR (3.9–10.0 mmol L^−1^) was improved from 61.4% in the first 24 h to 85.5% in the last 24 h of the treatment period (*P* = 0.03). Time spent above 13.9 mmol L^−1^ was decreased from 10.6% to 0.9%, and time spent above 10.0 mmol L^−1^ was decreased from 37.5% to 13.6%. Time spent below 3.9 mmol L^−1^ remained low throughout the trial, with 1.2% at the first 24 h and 1.3% in the last 24 h. In addition, glycemic variability was slightly decreased during the treatment period (coefficient of variation (CV) of 27.7% at the first 24 h and 22.6% at the last 24 h) (Extended Data Fig. 7b). No episodes of severe hypoglycemia (that is, requiring clinical intervention) or hyperglycemia with ketosis occurred during the trial.

Finally, physicians who participated in the trial were asked to complete satisfaction questionnaires on the AI system at end of each patient’s treatment intervention (Extended Data Fig. 7c). Most physicians stated that the AI interface is understandable (4.57/5.00), time-saving (4.50/5.00), effective (4.00/5.00) and safe (4.29/5.00) in routine clinical practice, with an overall satisfaction score of 4.14/5.00 (Fig. 6d).

## Discussion

In this study, we developed an RL-based AI system, called RL-DITR, for personalized and dynamic insulin dosing for patients with T2D. We performed development phase validation and clinical validations, including internal validation, comparing AI to physicians using quantitative and qualitative metrics, external validation with test–retest, prospective deployment with test–retest and a proof-of-concept feasibility study with clinical trial. Taken together, our findings demonstrate that our RL-DITR system has potential as a feasible approach for the optimized management of glycemic control in inpatients with T2D.

The management of blood glucose in diabetes remains challenging due to the complexity of human metabolism, which calls for the development of more adaptive and dynamic algorithms for blood glucose regulation. Conventional insulin titration relies largely on physicians’ experience, following the clinical guidelines. To address the challenge of personalized insulin titration algorithm for glycemic control, our RL-based architecture is tailored to achieve precise treatment for individual patients, with clinical supervision. First, we constructed a patient model, as an intermediate step, to provide knowledge of the environment’s dynamics (glucose dynamics) for a policy model. Our proposed patient model-based RL model can make multi-step planning to improve prescription consistency. In addition, because the multi-step plan can be interpreted as the intent of the model from now to a span of time period into the future, it offers a more informative and intuitive signal for interpretation^28^. Additionally, our RL-based system delivers continuous and real-time insulin dosage recommendation for patients with T2D who are receiving subcutaneous insulin injection, combining optimal policies for clinical decision-making and the mimicking of experienced physicians^29^.

Another strength of our study is that we conducted a comprehensive early clinical validation of the AI-based clinical decision-making system across various clinical scenarios. These validations, regarded as a standard of care and quality assurance review^30^, evaluate the AI system’s clinical performance and provide a basis for its effective integration from development to adoption into clinical practice. In clinical deployment, our AI framework offers potential benefits, including automated reading of a large number of inputs from the EHRs, integration of complex data and accessible insulin dosing interface. The user-friendly interface, designed to align with physicians’ workflow, gains increased willingness of adoption in routine clinical practice.

Although some algorithms have been developed to assist physicians in insulin titration, only a few have been validated in clinical trials^31,32^. We conducted a proof-of-concept feasibility trial demonstrating the viability of the RL-DITR system in inpatients with T2D. Notably, the use of the RL-DITR system resulted in a considerable improvement in blood glucose control, meeting our pre-determined feasibility goal. The percentage of well-controlled blood glucose levels of TIR also demonstrated a substantial increase. Managing hypoglycemia risk is a key consideration for real-world deployment of the AI system. While achieving improved control of blood glucose levels, the system did not increase the risk of hypoglycemia. Additionally, physicians using the RL-DITR system have reported an increased level of satisfaction, including aspects such as efficiency in clinical practice and perceived effectiveness and safety in glycemic control. These results suggest that our RL-DITR system has the potential to offer feasible insulin dosing to inpatients with T2D. A large and multi-center randomized controlled trial would help to determine the efficacy and benefits of this clinical AI solution. Our RL-DITR system was designed as a closed-loop intelligent tool that could use real-time patient data to track blood glucose trajectories and modify treatment regimens accordingly. With our ‘digital twin’ system for patients with T2D, it can be used to provide an on-demand risk profile (patient model) of hyperglycemia and hypoglycemia of T2D and offer treatment suggestions to mitigate the risks (policy).

Furthermore, the RL-DITR system was developed using EHRs of inpatients with T2D, but its generalizability to other populations, such as outpatients, needs further investigation. We conducted simulated experiments using Gaussian noise to mimic low data quality and dropout^33^ to simulate missing data scenarios before deployment (Supplementary Fig. 1). Additionally, we obtained a retrospective validation set (*n* = 27) of patients with diabetes from outpatient settings and conducted an assessment of our model, demonstrating that our model was able to provide recommendations under these conditions (Supplementary Table 1). Therefore, although the RL-DITR workflow was implemented and tested for inpatients with T2D, there exists the possibility to extend its application to a wider range of healthcare settings, such as outpatient management, given appropriate integration and continued development.

Although our RL-DITR system has achieved good performance in insulin dosage titration, some challenges remain. First, our data were collected from individuals of various ethnicities in China, predominantly Han Chinese (92%) as well as Hui, Uyghur, Mongol and so on. The generalization of the AI to other ethnicities needs to be further investigated. Second, the variety of diet during the hospitalized period was uniformly supplied in the EHRs to build our model. For patients out of hospital, dietary variation and physical activity should be taken into account and explored by our RL model. We have opened an interface to accumulate dietary information for late updated model.

In conclusion, we developed an RL-based clinical decision-making system for dynamic recommendation of dosing that demonstrated feasibility for glycemic control in patients with T2D. The RL-DITR system is a model-based RL architecture that could enable multi-step planning for patients with long-term care. With the integration of RL structure and supervised knowledge, the RL-DITR system could learn the optimal policy based on non-optimized data while retaining the safe states by balancing exploitation and exploration. Furthermore, we performed a stepwise validation of the AI system from simulation to deployment and a proof-of-concept feasibility trial. These demonstrate the RL approaches as a potential tool to assist clinicians, especially junior physicians and non-endocrine specialists, with diabetes management in hospitalized patients with T2D. Further studies are needed to investigate the AI’s generalizability to various scenarios, such as in the outpatient or primary care settings.

## Methods

### Study design and participants

To train and validate a computational clinical decision support model, we constructed a large multi-center dataset using EHRs of hospitalized patients with T2D who received insulin therapy from January 2013 to April 2021 in the Department of Endocrinology and Metabolism, Zhongshan Hospital and Qingpu Hospital, in Shanghai, China. After an exclusion of the patients who used insulin pumps or glucocorticoids or received insulin for fewer than 2 d during hospitalization, a total of 12,981 patients with T2D with 119,941 treatment days were included to develop and validate the RL-DITR model. The inpatients included in our study were patients in the Department of Endocrinology who received insulin treatment for glycemic control, without acute illness or procedures/surgery. The demographics and clinical characteristics of patients are presented in Extended Data Table 1, demonstrating a typical T2D population.

We conducted stepwise studies to evaluate the performance of our RL-DITR model (version 1.0) from development to early clinical evaluations: (1) retrospective study from the modeling development hospital (the internal cohort); (2) retrospective study from the hospital out of modeling development (the external cohort); and (3) a prospective test–retest review of AI plan/regimen after deployment (the prospective deployment study). In addition, we performed a proof-of-concept feasibility trial of the RL-DITR system in clinical practice with inpatients with T2D who were admitted for optimization of glycemic control at Zhongshan Hospital (ClinicalTrials.gov: NCT05409391) (details of proof-of-concept trial protocol provided in Supplementary Information). The retrospective study obtained the following institutional review board (IRB) approval: Zhongshan Hospital, Shanghai, China (2019-014R); XuHui Central Hospital, Shanghai, China (2021-007) and Qingpu Branch of Zhongshan Hospital, Shanghai, China (2021-25). Patient informed consent was waived by the Ethics Committee. The prospective study and proof-of-concept feasibility trial were approved by the Ethics Committee of Zhongshan Hospital, Fudan University. Each participant provided written informed consent for the prospective study and the proof-of-concept feasibility trial.

### Development and validation of the model-based RL system

#### Time-series data pre-process and NLP

For time-series data representation, every patient in the dataset was represented as a temporal sequence of feature vectors. Specifically, each day was broken into seven time periods, including pre-breakfast, post-breakfast, pre-lunch, post-lunch, pre-dinner, post-dinner and pre-bedtime. All records that occurred within the same period were grouped together and formed a feature set to feed into the RL model as input (detailed list of the input features provided in Supplementary Table 2). For structured data, we aligned and normalized them. For free-text notes, we applied a pre-trained language model, ClinicalBERT. Specifically, we first trained the ClinicalBERT on a large corpus of EHR data. ClinicalBERT is a masked medical domain language model that predicts randomly masked words in a sequence and, hence, can be transformed into downstream tasks. Then, the ClinicalBERT was fine-tuned for information extraction from free text.

We further automatically extracted temporal features from patient clinical records, including clinical observations (blood glucose records), a sequence of decision rules to determine the course of actions (for example, treatment type and insulin dosage titration) and clinical assessment of patients. The numerical values were extracted from demographics, laboratory reports, blood glucose and medications and further translated with standard units according to the LOINC database. Then, each numerical value was normalized to a standard normal distribution. In terms of discrete values, all the diagnoses of a patient were mapped onto the International Classification of Diseases-9 (ICD-9) and used as discrete features, encoded as binary presence features. We constructed a large multi-center dataset with a large corpus of 1.2 billion words of diverse diseases to train a ClinicalBERT pre-trained model. ClinicalBERT was fine-tuned on a multi-label dataset to extract 40 symptom labels from medical notes. Phenotype data were extracted from free-text notes of chief history of present illness and physical examination by ClinicalBERT. Validated on 1,000 annotated samples from the training set, the results showed that ClinicalBERT could accurately identify the symptom information with an average F1 score of 94.5%. Each extracted symptom label was encoded as a binary presence feature.

#### Building the computational model

The process of patient trajectory and treatment decision-making could be formulated as a Markov decision process (MDP). An MDP^34^ is a tuple (*S*, *A*, *P*, *G*, *γ*), where *S* and *A* are sets containing the states and actions, respectively; *P* is a transition function; *G* is a reward function; and *γ* is a discount factor. Given a dataset *D* = {*X*}, a trajectory $$X=\left\{\left({s}_{t},{a}_{t},{r}_{t}\right):t=1,\ldots ,\tau \right\}$$ shows each transition $$({s}_{t}$$,$${a}_{t},{r}_{t},{s}_{t+1})$$ from the step $$t$$ to the step *t* + 1, where *τ* is the length of the trajectory; $${s}_{t}$$ is a current state; $${{\rm{a}}}_{t}$$ is an action; $${{\rm{r}}}_{t}$$ is an immediate reward from $$G\left({{\rm{r}}}_{t}|{{\rm{s}}}_{t},{{\rm{a}}}_{t}\right)$$; and $${s}_{t+1}$$ is the next state after taking action $${{\rm{a}}}_{t}$$ from $$P\left({{\rm{s}}}_{t+1}|{{\rm{s}}}_{t},{{\rm{a}}}_{t}\right)$$. The goal of the MDP is to learn a policy model $$\pi ({a|s})$$ on the dataset $$D$$ to give treatment recommendations such that the cumulative reward $$\mathop{\sum }\nolimits_{t=0}^{T}{\gamma }^{t}{r}_{t}$$ representing clinical outcome is maximized.

To solve the MDP and obtain an effective and safe policy model $\pi$, we used a model-based RL approach, RL-DITR, which consisted of a patient model to track patients’ evolving states and a policy model to learn dynamic regimen strategy. The patient model was learned from historical trajectories, approximating the transition function *P* and the reward function *G* and providing support for policy model learning and planning. The policy model iteratively interacted with the patient model as an environment. At each step, the patient model generated state transition, status prediction and reward estimation based on observed patient trajectories. The policy model, taking the state as input, generated an action that was fed to the patient model. The patient model updated the states recurrently by an iterative process, enabling the policy model to plan for sequences of actions and find optimal solutions across generated trajectories.

#### Observation representation

To obtain states as input under the MDP setting, we first learn a representation function ${f}_{R}$, which maps past observations (for example, historical records of glucose levels) into a state space. Given an observation trajectory with $T$ steps ${O}_{1:T}=\left({o}_{1},{o}_{2},\,\ldots ,\,{o}_{T}\right)$, ${f}_{R}$ mapping ${O}_{1:T}$ to an initial hidden state $s$—that is, $s={f}_{R}({O}_{1:T})$, where *o*_*t*_ at step $t$ is a feature vector, including past information of demographics, diagnosis, symptom, medication, glucose level and laboratory test. The hidden state would be used as input for patient model and policy model.

#### Patient model for trajectory tracking

For patient trajectory tracking, we trained a patient model. The patient model consists of a dynamics function ${f}_{T}$ and a prediction function ${f}_{P}$. The ${f}_{T}$ was trained to map the current states ${s}_{t}$, action ${a}_{t}$ to the next state ${s}_{t+1}$ with a reward ${r}_{t}$—that is, ${s}_{t+1},{r}_{t}={f}_{T}\left({s}_{t},{a}_{t}\right)$, where *s*_1_ is obtained from past observations and derived from the representation function ${s}_{1}={f}_{R}\left({O}_{1:{t}^{{\prime} }}\right)$—and ${t}^{{\prime} }$ is the number of past steps. The prediction function ${f}_{P}$ takes an input of state ${s}_{t}$ and predicts patient status ${y}_{t}$—that is, ${y}_{t}={f}_{P}\left({s}_{t}\right)$. In this study, at each time $t$, ${y}_{t}$ includes a blood glucose value and a label of glucose within target range. The action ${{\rm{a}}}_{t}$ is a medication action of dosage decision of insulin, ranging from 1 to 40. The reward ${r}_{t}$ is the patient status score calculated based on glucose value for each measurement, according to the Magni risk function^35^ (Extended Data Fig. 2b):

$${risk}\left(b\right)=\left\{\begin{array}{l}\qquad\qquad\qquad\qquad\qquad\quad-1, \qquad b < 70\\ 1-\frac{{Cli}{p}_{0,15.5}\left(10* {\left({c}_{0}* {{\log }}{\left(b\right)}^{{c}_{1}}-{c}_{2}\right)}^{2}\right)}{7.75},\qquad\quad{else}\end{array},\right.$$

where *b* denotes the blood glucose level, and ${Cli}{p}_{{\epsilon }_{1},{\epsilon }_{2}}$ is a function that performs value clipping ${Cli}{p}_{{\epsilon }_{1},{\epsilon }_{2}}\left(x\right)={\rm{\min }}\{{\epsilon }_{2},{\rm{\max }}\{{\epsilon }_{1},x\}\}$, *c*_0_ = 1.509, *c*_1_ = 1.084 and *c*_2_ = 5.381. *r*_*t*_ ranges from −1 to 1. When conducting correlation analysis with daily outcome, Magni risk values were summed for each day.

We trained the dynamics function ${f}_{T}$ and the prediction function ${f}_{P}$ with joint learning. For the dynamics function ${f}_{T}$, we applied a consistency loss. Given an observation trajectory *O*_1:*T*_, we obtained a hidden state by the representation function ${f}_{R}$ and the dynamics function ${f}_{T}$ with $k$ steps, denoted as ${s}_{T,k}$. We proposed a consistency loss *L*_*T*_, where ${L}_{T}=\sum _{i,j}{\|{s}_{{T}_{i},{k}_{i}}-{s}_{{T}_{j},{k}_{j}}\|}_{2}$, where *T*_*i*_ + *k*_*i*_ = *T*_*j*_ + *k*_*j*_, *i* ≠ *j*, *L*_*T*_ is an MSE loss. The prediction function *f*_*P*_ was learned using SL ${L}_{P}(\,y,\hat{y})$, where the loss ${L}_{P}={L}_{P,{GLU}}+{L}_{P,{TIR}}$ based on ground truth $y$ and predicted $\hat{y}$ includes an MSE loss ${L}_{P,{GLU}}$ for learning glucose level prediction and a cross-entropy loss ${L}_{P,{TIR}}$ for a binary classification of WTR for each point. The total loss *L* = *μ*_*LT*_ + *L*_*P*_ was used for joint learning—here, $\mu$ is a weight. Both of the dynamics function *f*_*T*_ and the prediction function *f*_*P*_ shared the representation encoder *f*_*R*_ when training and inference. *f*_*R*_ was optimized together through backpropagation with the loss to capture meaningful patient representations and dynamics. To evaluate our hidden state embedding, we mapped the patients’ health states to low-dimensional projection using principal component analysis (PCA) (Extended Data Fig. 2a). Each node indicates the states of a patient. The state distribution demonstrated a good cluster hierarchy that individuals in the same cluster are associated with their observable properties (diabetes outcome, such as glucose level).

#### Policy model for insulin dosing

Based on a learned patient model, we could simulate the execution of the policy interacting with the patient model, allowing us to train a policy model $\pi$ to recommend insulin dosage. The *π* maps the current state *s*_*t*_ to the action ${a}_{t}$—that is, ${a}_{t} \sim \pi \left(\cdot |{s}_{t}\right)$. We combined the SL and RL to learn the policy model, with the expert supervision of safe actions to take into account. Specifically, we applied policy gradient optimization for training the policy model *π* to maximize the returned rewards while incorporating constrained supervision by expert experience.

For the SL part, we used the action made by the clinicians as supervision for policy update. An SL loss ${L}_{{SL}}({a}_{t},{\hat{a}}_{t})$ was set to minimize the difference between the action ${\hat{a}}_{t}$ recommended by the policy model *π*(*s*_*t*_) and the action ${a}_{t}$ made by the clinician. For the RL part, we optimized the policy model *π* based on the patient model (*f*_*T*_, *f*_*P*_) as an interactive environment, where a given trajectory was updated recurrently by an iterative process. For instance, at step $t$, given an action ${\hat{a}}_{t}$ recommended by the policy model *π*, the patient model maps from the current state ${s}_{t}$, action ${\hat{a}}_{t}$ to the next state ${s}_{t+1}$ with a reward ${\hat{r}}_{t}$. The policy model π received the updated state ${s}_{t+1}$ and proposed a new action ${\hat{a}}_{t+1}$. The policy model *π* was trained by both historical and obtained trajectories. Thus, the RL loss includes ${L}_{R{L}_{1}}=-\mathop{\sum }\nolimits_{t=1}^{T}{R}_{t}\log \pi ({a}_{t}|{s}_{t})$ and ${L}_{R{L}_{2}}=-\mathop{\sum }\nolimits_{t=1}^{T}{\hat{R}}_{t}\log \pi ({\hat{a}}_{t}|{s}_{t})$, where ${R}_{t}=\mathop{\sum }\nolimits_{i=0}^{T-t}{\gamma }^{i}{r}_{t+i}$ is the accumulated discount reward; $\gamma$ is the discount ratio; ${\hat{R}}_{t}$ is a value derived from the patient model; and $\pi ({a|}{s}_{t})$ represents the probability of taking action $a$. Therefore, a joint loss of SL and RL was optimized simultaneously: $L={L}_{R{L}_{1}}\left({a}_{t},{R}_{t}\right)+{\varepsilon }_{1}{L}_{R{L}_{2}}\left({\hat{a}}_{t},{\hat{R}}_{t}\right)+{\varepsilon }_{2}{L}_{{SL}}({a}_{t},{\hat{a}}_{t})$, where ${\varepsilon }_{1}$ and ${\varepsilon }_{2}$ are weights to tradeoff between ${L}_{{RL}}$ and ${L}_{{SL}}$. Of them, ${L}_{R{L}_{1}}$ and ${L}_{R{L}_{2}}$ enable the model to optimize its policy based on the clinical outcomes, such as glycemic values WTR, whereas the incorporation of ${L}_{{SL}}$ ensures that the generated actions align with clinically feasible ranges. The additional RL loss function ${L}_{R{L}_{2}}$ is designed to enhance the model’s performance and adaptability by learning from an extensive set of trajectories generated from the patient model. For stably training, following previous work^36^, a learnable value function $V\left(s\right)$, an expected return starting from $s$, was used instead of $R$.

With the dynamics function *f*_*T*_, the policy model can generate a K-step plan consisting of a sequence of K actions to perform in the next few steps in turn—for example, *K* = 7 for the daily treatment of the next day. Specifically, given a hidden state ${s}_{t}$, for $i\in \,[0,K-1],$ we sample ${a}_{t+i} \sim \pi ({\cdot |}{s}_{t+i})$ by the policy model. The next hidden state ${s}_{t+i+1}$ is then derived given the previous hidden state ${s}_{t+i}$ and the generated action ${a}_{t+i}$— that is, ${s}_{t+i+1},{r}_{t+i}={f}_{T}\left({s}_{t+i},{a}_{t+i}\right)$ by the patient model. This recurrent process runs iteratively until the condition of $K$ steps is reached. The plan value of the trajectory of $K$ steps is defined as $v=\mathop{\sum }\nolimits_{i=0}^{K-1}{\gamma }^{i}{r}_{t+i}+{\gamma }^{K}V\left({s}_{t+K}\right)$ (ref. ^37^), and the treatment plan of actions was derived by $\mathop{{{\max }}}\nolimits_{i}v\left({s}_{t},{a}_{i,t},{a}_{i,t+1},\ldots ,{a}_{i,t+K-1}\right)$. We applied a beam search for policy search^38^. The top B highest-value trajectories were stored at each timestep, where B was the beam size.

#### Training process

The training process involved two stages to optimize the models of our AI system (Extended Data Fig. 1b). During the first stage, we trained a patient model, including a dynamics function ${f}_{T}$ and a prediction function ${f}_{P}$. These functions were jointly optimized through the loss for state transitions and the loss for status prediction. Next, we learned a policy model $\pi$ by using a combined approach of both SL and RL with the trained patient model. The policy model was trained through a joint optimization process, minimizing both a policy gradient loss on trajectories and a supervised loss that constrains the difference between the recommended action from the policy model and the action taken by the clinician.

We used a transformer-based network with three layers as the representation function used to represent the observations of time-series data, as it has been shown to enable capturing the long dependence in the temporal information of patients^39^. The last hidden vector of the output hidden vectors was used for the initial state. We also applied a transformer network with three layers for dynamics function. The hidden dimension was set to 256, and the number of multi-attention heads was set to 8. We used three-layer multi-layer perceptrons (MLPs) for prediction function, policy function and value function. The hidden dimension was set to 256. The discount factor $\gamma$ was set to 0.9. Training of models by back-propagation of errors was performed in batches of 32 trajectories with padding to length of 128 for 100 epochs with a learning rate of ${10}^{-3}$. Training was performed using the Adam optimizer with a weight decay of ${10}^{-4}$. The weights $\mu$ and $\varepsilon$ were set to 0.1 and 1.0, respectively. The beam size B was set to 10. Dropout with $p=0.4$ was applied during training to improve and generalize network learning. The models were implemented using PyTorch.

#### Policy evaluation and simulation study

We applied policy evaluation to assess the value of a given learned policy using patient trajectories generated from the clinicians’ policy—given the behavior policy ${\pi }_{0}$ (the clinicians), from which actual patient trajectories were generated, and target policy ${\pi }_{1}$ (AI model). The importance sampling for policy evaluation was performed, which enables the evaluation of a target policy using data collected from a distinct policy^34^. Given $\left({s}_{t},{a}_{t},{r}_{t}\right)$ at time $t$, the importance sampling ratio at each step is defined ${\rho }_{t}={\pi }_{1}\left({a}_{t}|{s}_{t}\right)/{\pi }_{0}({a}_{t}|{s}_{t})$; the weight of the trajectory is $w={\prod }_{t=1}^{T}{\rho }_{t}$; and the estimated value is ${V}_{{IS}}$ = ${\sum }_{i=1}^{N}\tfrac{{w}_{i}}{N}{\sum }_{t=1}^{{T}_{i}}{\gamma }^{t-1}{r}_{i,t}$. To enhance the numerical stability of the calculations, we employed WIS along with effective sample size^40,41^, which normalizes the trajectories, thereby reducing variance^27^. The weights are calculated as ${w}_{i}={\rho }_{i,{T}_{i}}/{\sum }_{j=1}^{N}{\rho }_{j,{T}_{j}}$, and the estimated value is ${V}_{{WIS}}$ = ${\sum }_{i=1}^{N}{w}_{i}{\sum }_{t=1}^{{T}_{i}}{\gamma }^{t-1}{r}_{i,t}$.

To explore the model’s performance under various data conditions, we designed a simulation study. Given specific data conditions, such as no more than k blood glucose measurements per day, we randomly discard blood glucose values within the trajectories to ensure that the remaining trajectories satisfy this criterion.

#### Traditional insulin dosing method

The traditional clinical methods of insulin dosage titration were used as the standard clinical methods for comparison, consisting of guidelines^42^ and consensus formulas^43,44^ for premixed insulin regimen, basal regimen and basal-bolus regimen.

The insulin dosage titration rules of biphasic/premixed insulin regimen were as follows: pre-breakfast insulin dosage was adjusted based on the pre-supper glucose value; and pre-supper insulin dosage was adjusted based on the pre-breakfast glucose value. The detailed adjustment was according to the following formula:

$$f\left(x\right)=\left\{\begin{array}{c}-2,\,x < 80\\ 0,\,80\le x\le 109\\ 2,\,110\le x\le 139\\ \,4,\,140\le x\le 179\\ 6,\,x\ge 180\end{array}\right.$$

where *x* represents the corresponding blood glucose (mg dl^−1^), and *f*(*x*) represents the insulin adjusted dosage.

The insulin dosage titration rules of basal-only (usually long-acting insulin) regimen was as follows: pre-breakfast/bedtime basal insulin dosage was adjusted based on pre-breakfast/fasting glucose value. The detailed adjustment was according to the following formula:

$$f\left(x\right)=\left\{\begin{array}{c}-2,\,x < 80\\ 0,\,80\le x\le 109\\ 2,\,110\le x\le 139\\ \,4,\,140\le x\le 179\\ 6,\,x\ge 180\end{array}\right.$$

where *x* represents the pre-breakfast/fasting glucose (mg dl^−1^), and *f*(*x*) represents the basal insulin adjusted dosage.

The insulin dosage titration rules of basal-bolus regimen were as follows. If the fasting or mean blood glucose during the day was >140 mg dl^−1^ in the absence of hypoglycemia, basal (usually long-acting insulin) dosage was increased by 20% every day. If the patient developed hypoglycemia (<70 mg dl^−1^ ${\rm{mg}}/{\rm{dl}}$), basal insulin dosage was decreased by 20%. Also, bolus insulin dosage (usually short/rapid-acting insulin) was adjusted based on post-meal glucose value, according to the following formula:

$$f\left(x\right)=\left\{\begin{array}{l}4, \qquad 141\le x\le 180\\ 6, \qquad 181\le x\le 220\\ 8, \qquad 221\le x\le 260\\ 10, \quad \,\,\, 261\le x\le 300\\ 12, \quad \,\,\, 301\le x\le 350\\ 14, \quad \,\,\, 351\le x\le 400\\ 16, \quad \,\,\, x > 400\end{array}\right.$$

where *x* represents the corresponding post-meal glucose (mg dl^−1^), and *f*(*x*) represents the supplemental bolus insulin dosage.

### Study design for performance comparison of AI versus physicians

The performance of AI-generated insulin regimens was evaluated in three independent phases: (1) retrospective study from the modeling development hospital (the internal cohort); (2) retrospective study from the hospital out of modeling development (the external cohort); and (3) a prospective test–retest review of AI plan/regimen after deployment (the prospective deployment study).

Retrospective study phase of the internal cohort. Forty eligible patients with T2D treated with insulin injection were randomly selected from the retrospective EHRs of one of the modeling development hospitals (Qingpu Hospital) from May 2021 to December 2021. Two treatment days were randomly selected for each patient, resulting in 80 cases with 226 insulin points (Extended Data Fig. 4a). Three physician groups with different levels of clinical experience provided their dose recommendations, and the AI also generated insulin dose recommendations in silico for further evaluation. An expert consensus panel of three endocrinology specialists conducted blinded review and provided their own recommended insulin dosage. This was used as a reference insulin dosage for each insulin point to assess the accuracy of AI-generated dosage versus the three physician groups. The estimated acceptability, effectiveness and safety of the AI plan and the three physician groups’ plans were separately evaluated by the expert panel. The physician groups included junior (*n* = 5), intermediate (*n* = 5) and senior (*n* = 5) physicians with 1–3 years, 4–7 years and 8–20 years of clinical experience, respectively. Three endocrinology specialists who constituted the expert consensus panel were invited from the academic hospital, Zhongshan Hospital, and all were board certified and had at least 15 years of clinical experience.

Retrospective study phase of the external cohort. The retrospective dataset was collected from a non-teaching hospital (XuHui Hospital), which included 45 eligible consecutive patients with T2D from April 2021 to August 2021 (Extended Data Fig. 4b). The dataset contained 796 insulin points from 338 cases, and AI-generated dosage was compared to previously delivered insulin dosage by treating physicians (human plan) for accuracy evaluation. Next, we randomly selected 40 cases from the dataset to evaluate the acceptability, effectiveness and safety of the AI plan and the previous human plan. The evaluations were blinded head-to-head comparisons of AI versus human plans by the expert consensus with three independent experts. The same review was repeated after a minimum of 2 weeks to ensure reproducibility.

Prospective deployment study phase. AI was deployed (integrated) in Zhongshan Hospital beginning in November 2021 for real-time patient information read to output insulin dosage regimen in the doctor’s advice interface (Extended Data Fig. 5a). In May 2022, 40 consecutive AI-generated plans were tested for acceptance, effectiveness and safety by endocrinology physicians at the bedside (Extended Data Fig. 5b). After determining clinical adoption and ensuring adherence to standard clinical quality controls, the AI insulin regimen was used for patient treatment. The same review was retested by the physicians after an interval (>2 weeks) of day-to-day work with human–machine interaction. Three board-certified endocrinology physicians with clinical experience ranging from 5 years to 15 years participated in the test–retest review.

#### Case inclusion and exclusion criteria

The inclusion and exclusion criteria for patients were consistent across the three phases. Inclusion criteria were patients with T2D treated with subcutaneous insulin injection for at least two consecutive days. Patients with acute complications of diabetes, such as ketoacidosis or hyperglycemic hyperosmolar state, or patients who were treated with glucocorticoids, were excluded.

#### Definitions of the performance metrics

1. Quantitative evaluation. We used the metrics of MAE and agreement percentage to quantitatively evaluate the accuracy performance of insulin regimens. MAE represents the errors between predicted values and consensus values. The agreement was calculated by the difference of predicted value and consensus values and grouped as the following categories: (1) identical agreement: the adjustment direction given by the AI or human physician is consistent with the reference regimen, and the adjusted dosage was identical; and (2) clinical agreement: the adjustment direction given by the AI or human physician is consistent with the reference regimen, and the difference of dose was within 20%.
2. Acceptability. In the retrospective simulation study phases, questionnaires 2 and 3 (item 3) and questionnaire 4 (item 4) were used to ask the reviewers whether the insulin regimen was ‘acceptable’ or ‘unacceptable’ in clinical settings according to their judgment (Supplementary Information).
3. Effectiveness and safety. To evaluate the AI’s effectiveness, we used questionnaires 2 and 3 (item 4) and questionnaire 4 (item 5), which asked the reviewers if the recommended insulin regimen was perceived to bring glucose within the target range according to their judgment. The effectiveness was scaled on a five-point Likert scale ranging from 1 (very poor control of glycemia) to 5 (very good control of glycemia). For safety evaluation, we used questionnaires 2 and 3 (item 5) and questionnaire 4 (item 6), which asked the reviewers if the recommended insulin regimen was perceived to lead to an increased risk of hypoglycemia according to their judgment. The safety was then scaled on a five-point Likert scale ranging from 1 (very high risk) to 5 (very low risk) (Supplementary Information).
4. Superior plan. In the head-to-head comparison of the AI and human plans in the retrospective simulation study of the external cohort, the one (AI or human) selected as most clinically appropriate by the expert consensus review was considered as the superior plan (Supplementary Information).
5. Adoption. In the prospective deployment phase, the AI plans were reviewed by the endocrinology physicians at the bedside; the clinical adoption was determined; and the deemed AI insulin regimen was used for patient treatment following all standard clinical quality controls.

### Proof-of-concept feasibility trial

#### Trial design and participants

We conducted a proof-of-concept trial (ClinicalTrials.gov: NCT05409391) to evaluate the feasibility and safety of AI in inpatients with T2D from 28 June to 6 October 2022. This trial was a patient-blinded and single-arm intervention, which was performed in the ward of the Department of Endocrinology and Metabolism, Zhongshan Hospital, in China. The RL-DITR system was embedded in the insulin dosing interface of the health information system (HIS), allowing real-time reading of patient clinical information and insulin dosage regimen recommendation (Extended Data Fig. 5a). The healthcare provider could review the AI-generated insulin regimen and either ‘adopt’ or ‘reject’ it. An example of the AI recommendation report for the healthcare provider is presented in Extended Data Fig. 5a. Patients with T2D receiving subcutaneous insulin treatment were recruited and screened for the inclusion and exclusion criteria. Inclusion criteria were adults aged 18–75 years and HbA1c of 7.0–11.0%. The following patients were excluded: patients with body mass index ≥45 kg m^−^^2^; patients with ketoacidosis or hyperglycemic hyperosmolar state; patients with severe edema or peripheral vessel disorders; patients with surgery scheduled during hospitalization; or women who were pregnant or breast-feeding (details of the clinical trial protocol provided in Supplementary Information; CONSORT-AI checklist provided in Supplementary Table 3).

#### Trial intervention and glucose monitoring

The pre-intervention initial insulin regimen served as reference for daily insulin regimen. Eligible patients received insulin dosage titration according to the AI model after the first cycle of insulin regimen, which was confirmed twice daily by the physician in charge. The treating physician could reject the recommendation if deemed necessary. Each patient was studied for up to 5 d or until discharge from hospital. Throughout the trial, anti-hyperglycemic drugs remained unchanged; standard meals at usual mealtime were provided; and no physical activity was scheduled.

Capillary glucose concentration was measured at seven timepoints of fasting, after breakfast, before and after lunch, before and after dinner and before bedtime a day by a glucometer (Glupad, Sinomedisite) to estimate glucose control and to guide insulin regimen. The goal was to achieve preprandial capillary blood glucose of 5.6–7.8 mmol L^−1^ and postprandial capillary glucose of less than 10.0 mmol L^−1^ (ref. ^5^). CGM (Abbott FreeStyle Libre) was also used for each patient to estimate the percentage of continuous glucose measurements within/above/below range (3.9–10.0 mmol L^−1^). The CGM data were analyzed by physicians, and the treatment was not influenced by data gained by CGM. CGM alarms were not activated during the feasibility clinical trial.

#### Outcomes

The primary outcome was difference in glycemic control as measured by mean daily blood glucose concentration (total, preprandial and post-prandial capillary blood glucose). The secondary endpoints included glucose concentration in the target range (TIR) of 3.9–10.0 mmol L^−1^, glucose concentration above range (10.1–13.9 mmol L^−1^ or >13.9 mmol L^−1^) or below range (3.0–3.8 mmol L^−1^ or <3.0 mmolL^−1^) and glycemic variability. Glycemic variability was determined by the CV of glucose values. Safety was assessed as the number of hypoglycemic events. Serious adverse events included severe hypoglycemia, defined as a capillary glucose level of less than 2.2 mmol L^−1^ or an episode that required the assistance of another person, and hyperglycemia (>20 mmol L^−1^) with ketonemia or hyperosmolar coma, along with other serious adverse events.

#### Statistical analysis

The sample size calculation was based on the primary outcome. According to the literature^44^ and data from previous diabetic inpatients at Zhongshan Hospital, the mean daily capillary blood glucose was estimated to be 11 mmol L^−1^ at baseline and reduced by the standard difference of 2.5 mmol L^−1^ after insulin optimization by clinicians^44–46^. With a power of 90% and one-sided α = 0.025, 13 participants were required. Considering a 20% loss to follow-up, 16 participants were estimated to be required. PASS software version 11.0 was used to calculate the sample size. Clinical studies were analyzed using SAS 9.3 software, and a two-sided *P* value of less than 0.05 was considered statistically significant. The matched *t*-test was used to compare the performance of RL-DITR and physicians. The change from baseline measurements to the end of the trial was analyzed by two-sided paired *t*-test and a Wilcoxon signed-rank test for continuous measurements. The seven-point blood glucose profiles were analyzed using a generalized linear mixed model. The model used a Noisy-OR approach to aggregate WTR predicted probabilities of points for daily WTR prediction.

### Reporting summary

Further information on research design is available in the Nature Portfolio Reporting Summary linked to this article.

## Online content

Any methods, additional references, Nature Portfolio reporting summaries, source data, extended data, supplementary information, acknowledgements, peer review information; details of author contributions and competing interests; and statements of data and code availability are available at 10.1038/s41591-023-02552-9.

### Supplementary information

Supplementary Information Supplementary Information, Fig. 1 and Tables 1–3.

Reporting Summary

## Figures and Tables

**Extended Data Table 1:**
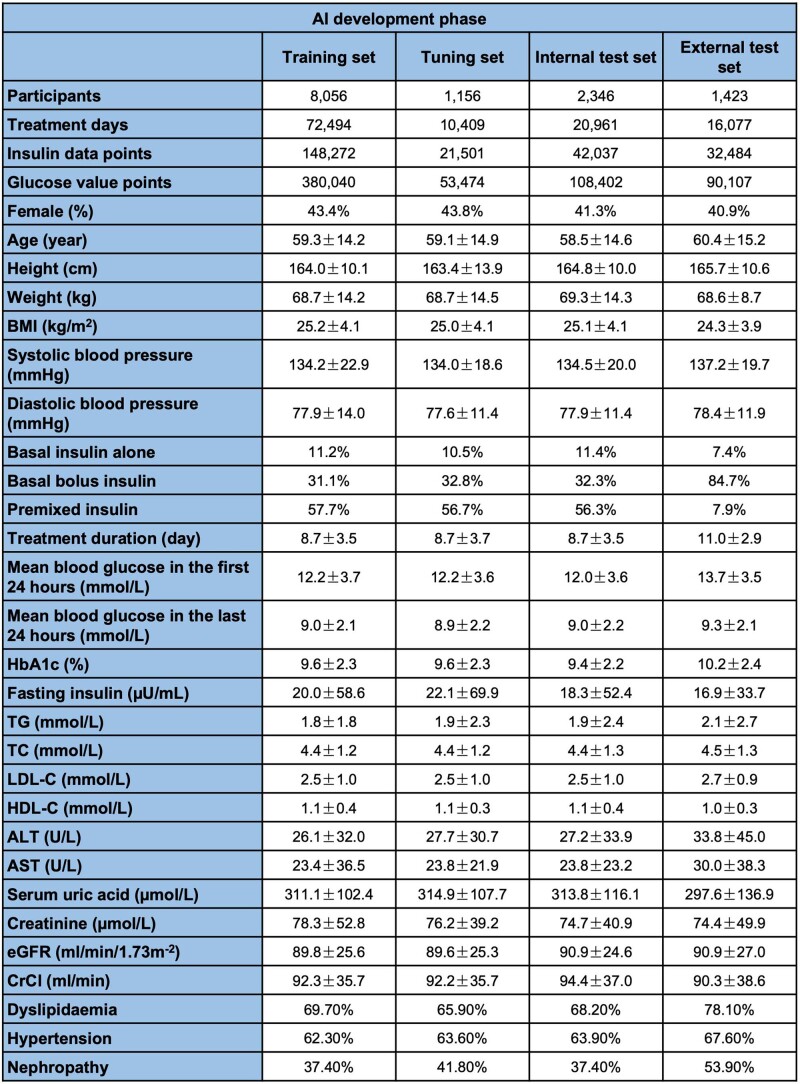
Baseline characteristics of patients with T2D in the developmental dataset and internal and external test sets for treatment regimen in the AI development phase

**Extended Data Table 2:**
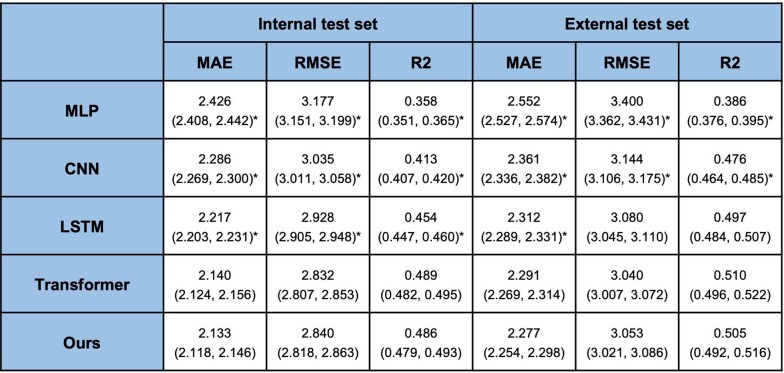
Performance comparison of patient model versus other methods for glucose prediction in internal and external test sets in the AI development phase

Each column represents the evaluation metrics. RMSE, root mean square error. Parentheses indicate 95% CIs. Significance levels are denoted as * for *P* < 0.05, where *P* values were computed using one-sided permutation tests with 1,000 resamples.

**Extended Data Table 3:**
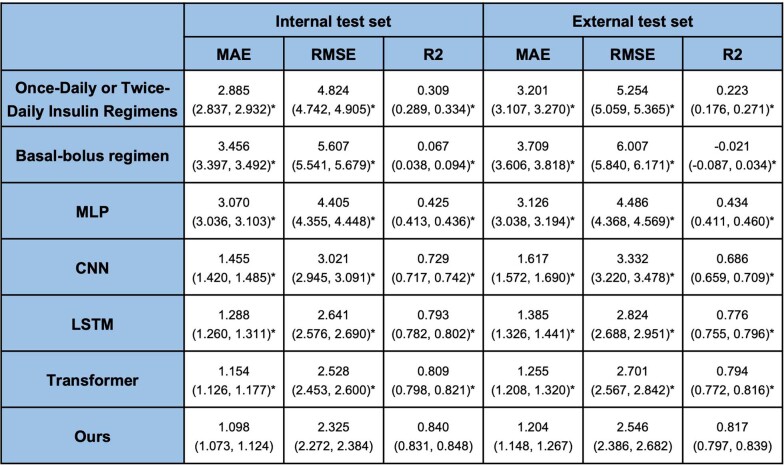
Performance comparison of our RL-based model versus other SL-based methods and traditional regimens for insulin titration regimens in internal and external test sets in the AI development phase

Each column represents the evaluation metrics. RMSE, root mean square error. Parentheses indicate 95% CIs. Significance levels are denoted as * for *P* < 0.05, where *P* values were computed using one-sided permutation tests with 1,000 resamples.

## Data Availability

IRB approval was obtained from institutions for EHR data collection. Individual-level patient records can be accessible with IRB consent and are not publicly available. De-identified data can be requested by contacting the corresponding authors. All data access requests will be reviewed and (if successful) granted by the Data Access Committee. Data can be shared only for non-commercial academic purposes and will require a formal material transfer agreement. Generally, all such requests for access to EHR data will be responded to within 1 month. For the reproduction of our code and model, we have also deposited a minimum dataset at Zenodo (https://zenodo.org/record/8198049), which is publicly available for scientific research and non-commercial use. Individual-level data of the clinical trial (ClinicalTrials.gov: NCT05409391) reported in this study are not publicly shared. Data can be available to bona fide researchers for non-commercial academic purposes and necessitate a data user agreement. Requests should be submitted by emailing the corresponding authors (Y.C. or G.W.) at chen.ying4@zs-hospital.sh.cn or guangyu.wang24@gmail.com. All requests will be reviewed by the study’s steering committee to determine whether the data requested are subject to patient privacy obligations. Requests will be processed within a 2-week timeframe. All data shared will be de-identified.

## Code availability

The deep learning models were developed and deployed in Python (version 3.8) using PyTorch (1.9.0). The following standard model libraries were used: scikit-learn (1.0.1), pandas (l.3.4), numpy (l.20.3), matplotlib (3.5.0), pytorch-lightning (1.8.3) and transformers (4.28.0). Custom codes were specific to our development environment and used primarily for data input/output and parallelization across computers and graphics processors. The codes are available for scientific research and non-commercial use on GitHub at https://github.com/rlditr23/RL-DITR. The pre-trained model ClinicalBERT is publicly available (https://huggingface.co/medicalai/ClinicalBERT).
