# Supplementary material for: Optimized glycemic control of type 2 diabetes with reinforcement learning: a proof-of-concept trial
Source: Nat Med. 2023 Sep 14;29(10):2633–42. doi: 10.1038/s41591-023-02552-9 (PMC10579102; doi:10.1038/s41591-023-02552-9)
Supplement: Supplementary file 1 — Supplementary Information, Fig. 1 and Tables 1–3. [file 41591_2023_2552_MOESM1_ESM.pdf]

nature medicine

Article

https://doi.org/10.1038/s41591-023-02552-9

Optimized glycemic control of type 2 diabetes with reinforcement learning: a proof-of-concept trial

In the format provided by the authors and unedited

Supplementary Information

Table of Contents

Supplementary Information. Proof-of-concept trial protocol 2

Supplementary Information. Questionnaires 23

Supplementary Figure 1. Outpatient simulation study of our RL-based model. 34

Supplementary Table 1. Performance evaluation of our RL-based model in outpatient set 35

Supplementary Table 2. List of input features 36

Supplementary Table 3. Consort-AI checklist 40

Supplementary Information. Proof-of-concept trial protocol

Table of Contents

1. Background 3

2. Study Objectives 5

3. Study Design 7

4. Safety and Adverse Events 12

5. Participant Termination 15

6. Statistical Analyses 16

7. Ethical Requirement 18

8. Study Confidentiality 19

9. Training of Research Staff 20

10. Reference 21

Chapter 1

Background

1. Background

Type 2 diabetes (T2D) is prevalent and poses a great burden in the world. Data from the International Diabetes Federation (IDF) showed that the number of diabetic patients had reached 451 million in 2017, and it will rise to 693 million by 2045[1]. While good glycemic control can markedly reduce diabetic complications and mortality in diabetic patients[2,3]. Many T2D patients require insulin therapy for glycemic control in the course of disease progression. However, two-thirds of insulin users sustain glycated hemoglobin (HbA1c) levels above 7.0% (53 mmol/L), and more than a third sustain HbA1c levels higher than 9.0% (75 mmol/L) mainly due to inaccurate and inertia titration of insulin dosage[4].

Effective and safe insulin therapy is essential for glycemia management in T2D patients. The glycemic targets varied with the amounts and timing of dietary intake, physical exercise and anti-diabetic medication[5]. Moreover, conventional insulin therapy has the risk of hypoglycemia, which is also associated with increased morbidities and length of hospital stay[6]. Therefore, personalized and frequent dosage titrations are required to overcome constant variations in insulin requirements. The glycemia management in hospitalized patients has long been hindered by the need for experienced physicians to make frequent insulin dosage titration. Consequently, glycemic control of inpatients is often inadequate. To achieve this goal, an accurate a real-time insulin dosage titration system is needed at an individual level. Of note, current tools are mostly created based on general guidelines and cannot meet the need for clinicians to precisely titrate insulin dosage[7].

Artificial intelligence (AI) approaches have emerged as potentially powerful tools to aid in disease diagnosis and management[8-10], mimicking and perhaps even augmenting physicians in clinical decision-making. AI has been applied in image recognition of diabetic retinopathy and risk prediction for diabetes[11]. The recent promising work on EHRs-derived modelling[12] suggests that the incorporation of machine learning may enable clinical decision of insulin dosage titration.

In the current study, we have developed and evaluated a Reinforcement

Learning-based Dynamic Insulin Titration Regimen for T2D (RL-DITR) framework, which iteratively generates patient state trajectories with a patient model and learns the optimal treatment regimen by analyzing the reward from interacting with the patient environment, achieving real-time intelligent and personalized insulin titration for optimal treatment by mining the experience of clinicians with the combination of supervised learning and the RL. Furthermore, we conduct a proof-of-concept trial to evaluate the feasibility and safety of RL-DITR system on glycemic control of T2D patients who received subcutaneous insulin therapy.

Chapter 2
Study Objectives

2. Study Objectives

The objective of the study is to evaluate the performance of the model-based RL system in a real-world clinical setting in the therapeutic realm. Therefore, we conducted the prospective clinical intervention trial to evaluate the feasibility and safety of insulin dosage titration by RL-DITR system in glycemic control in T2D patients treated with subcutaneous insulin injection. We hypothesize that T2D patients receiving insulin dosage titration could achieve improving glycemic control with the assistance of RL-DITR system recommendation during the trial. The goal of insulin therapy was to achieve preprandial capillary blood glucose between 5.6-7.8 mmol/L and postprandial capillary glucose less than 10.0 mmol/L[13-15].

2.1 Primary Objectives

To evaluate the effect of insulin dosage titration according to RL-DITR system assessed by difference in glycemic control measured by mean daily capillary blood glucose concentration (total, preprandial, post-prandial capillary blood glucose).

2.2 Secondary Objectives

(1) To evaluate the feasibility of the insulin dosage titration recommended by RL-DITR system. The preprandial capillary glucose concentration and post-prandial capillary glucose concentration at each defined time are assessed during the trial period in T2D patients with insulin therapy.

(2) To evaluate the effect of insulin dosage titration according to RL-DITR system on glycemic control measured by the CGM. The percentage of time of sensor glucose concentration/capillary blood glucose in target range (TIR) of 3.9-10.0 mmol/L, above range (10.1-13.9 mmol/L or >13.9 mmol/L) or below range (3.0-3.8 mmol/L or <3.0 mmol/L) are assessed during the trial period in T2D patients with insulin therapy.

(3) To evaluate the effect of insulin dosage titration according to RL-DITR system on the mean sensor glucose concentration, glucose management indicator (GMI), and glycemic variability (%CV) during the trial period in T2D patients with insulin therapy.

(4) To evaluate the risk of hypoglycemia of insulin dosage titration according to RL-DITR system in T2D patients with insulin therapy.

Chapter 3

Study Design

3. Study Design

3.1 Design Overview

The study is an open-label, one-arm intervention trial to evaluate the feasibility and safety of RL-DITR system in clinical application. After screening for the inclusion and exclusion criteria, eligible patients will receive insulin dosage titration set with the assistance of RL-DITR recommendation system during the intervention trial. The goal of insulin therapy was to achieve preprandial capillary blood glucose between 5.6-7.8 mmol/L and postprandial capillary glucose less than 10.0 mmol/L. This study is conducted in the ward of the Department of Endocrinology and Metabolism, Zhongshan Hospital, China. All patients are studied for 5 consecutive days or until hospital discharge. For each patient, capillary glucose concentration was measured at 7-time points a day including, after breakfast, before and after lunch, before and after dinner, and before bedtime a day by Glucometer (Glupad, Sinomedisite, China). Capillary glucose measurements were performed by the nurse staff according to standard procedures with a point-of-care testing device, which is integrated into the HIS system. And continuous glucose monitoring (CGM) was performed using flash glucose monitoring (Abbott Freestyle Libre, USA) placed on the upper left arm. The CGM data were analyzed retrospectively, and the treatment was not influenced by data gained by CGM.

3.2 Study Population

Eligible patients should meet the inclusion criteria and be excluded from the study if they meet any of the exclusion criteria.

3.2.1 Inclusion criteria

Patients included in this study should meet the following criteria:

① Patients with type 2 diabetes;

② Aged 18-75 years old;

③ Insulin has been used for at least 1 months;

④ HbA1c: 7.0%-11.0%.

3.2.2 Exclusion criteria

Patients will be excluded from this study if they meet any of the following exclusion criteria:

① Acute diabetic complications including diabetic ketoacidosis or hyperglycemic hyperosmolar state;

② BMI≥45 kg/m^2;

③ Women who are pregnant or breast-feeding;

④ Patients with severe cardiac, hepatic, renal diseases;

⑤ Patients with any psychiatric or psychological diseases;

⑥ Patients with severe edema, infections or peripheral circulation disorders;

⑦ Patients who cannot comply with the protocol.

3.3 Intervention

The RL-DITR system take a patient's temporal sequence of feature vectors from real-world as input, and then provide dynamic and personalized insulin dosing regimens. Capillary glucose measurements were conducted by the nurse staff in charge and automatedly uploaded into the HIS system. The recommendation of insulin dosage reviewed by the healthcare providers via the Doctor's order interface. The healthcare provider can review the current recommendation and choose "adopt" or "reject". In the case of "reject", the healthcare providers can give feedback of the AI system in the log file.

Eligible patients receive RL-DITR intervention after enrollment. The patients receive one of three insulin regimens: long-acting insulin only ± antidiabetic drugs; biphasic or premixed insulin ± antidiabetic drugs; basal-bolus therapy ± antidiabetic drugs. The initial insulin dose is referred to the pre-study total daily insulin dose. After the first circle of insulin regimen, eligible patients receive insulin dosage titration according to the RL-DITR system recommendation, which has to be confirmed twice daily by the physician in charge. For each patient, capillary glucose concentration is measured at 7-time points of fasting, after breakfast, before and after lunch, before and after dinner, and before bedtime a day using Glucometer (Glupad, Sinomedisite, China). All patients are monitored for 5 consecutive days or until hospital discharge.

Continuous glucose monitoring device is used for sensor glucose measurement during the trial period. All patients are equipped with the flash glucose monitoring (Abbott Freestyle Libre, USA) on the upper left arm. Throughout the trial, the patients are provided standard meals at usual meal times in the ward. Participants remain largely sedentary during the trial, and no physical activity is scheduled. Anti-diabetic medications are not changed during the period of the study.

3.4 Study Procedure

The study consists of the following periods: 1) screening period before admission (<2-weeks); 2) run-in period on the first day of hospitalization; and 3) 5-days intervention period during hospitalization (Figure 1).

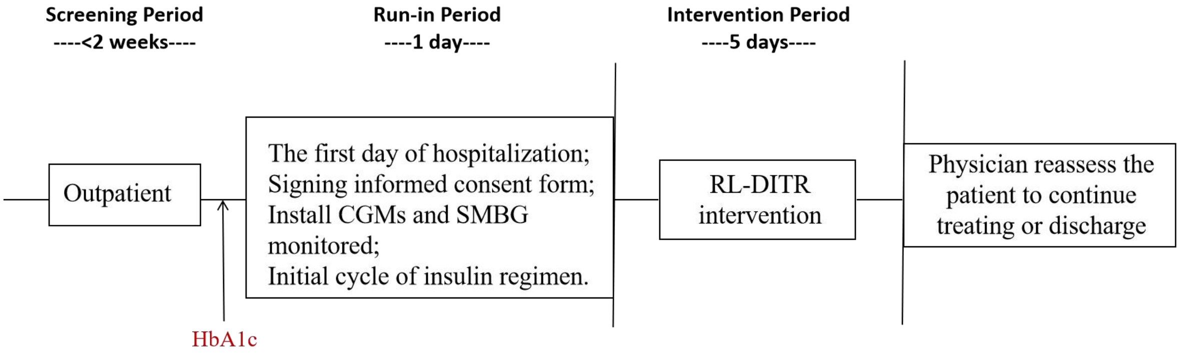

Figure 1. Flow chart

3.4.1 Screening period

T2D patients receiving insulin therapy will be eligible to be screened in the study. The information about the study will be provided to patients in oral and in writing. Written informed consent for participation in the study is obtained before performing any study-specific screening tests or evaluations. After signing the informed consent form, patients will be assigned a screening number and assessed for eligibility. Clinical characteristics and blood HbA1c level are evaluated at screening period. Screening period will be completed until admission.

3.4.2 Run-in period

The first day of hospitalization is the run-in period. Patients are reassessed for eligibility with regard to HbA1c results. Height, body weight, HbA1c level, and total pre-trial daily insulin dose are recorded. In the run-in period, the initial insulin regimen and dose are adopted as the same as that before admission. The RL-DITR system

fetches the patient's clinical information and initial insulin regimen and dosage and then gives the recommendation of the next insulin dose. All obligatory assessments should be completed in this period. The data obtained from clinical evaluation and laboratory tests for screening are used for baseline assessment as well.

3.4.3 Intervention period

3.4.3.1 Intervention

The RL-DITR system is a model-based clinical decision support system for insulin titration, which iteratively generates patient state trajectories with a patient model and learns the optimal treatment regimen by analyzing the reward from interacting with the patient environment, achieving real-time intelligent and personalized insulin titration for optimal treatment by mining the experience of clinicians with the combination of supervised learning and the RL. In the intervention period, insulin dosage is modified and titrated according to the RL-DITR recommendation. The initial insulin dose is referred to the pre-study total daily insulin dose. After the first circle of insulin regimen, eligible patients receive insulin dosage titration according to the RL-DITR system recommendation, which has to be confirmed twice daily by the physician in charge. To assess compliance, the RL-DITR recommendations adopted by physicians are documented and any deviation is recorded.

3.5 Study Outcomes

3.5.1 Primary outcomes

The primary outcome was difference in glycemic control as measured by the mean daily capillary blood glucose (total, preprandial, post-prandial capillary blood glucose) during the trial period (from the first 24h to the last 24h of the trial).

3.5.2 Secondary outcomes

1. The mean daily pre-prandial capillary blood glucose, and mean daily postprandial capillary blood glucose during the trial period (from the first 24h to the last 24h of the trial);
2. The difference of seven-point blood glucose profile between the first 24h and the last 24h of the trial;
3. The percentage of time of sensor glucose measurement/capillary blood glucose

in range (3.9-10.0 mmol/L) during the trial period;

4. The percentage of time of sensor glucose measurement/capillary blood glucose above range (10.1-13.9 mmol/L) during the trial period ;
5. The percentage of time of sensor glucose measurement/capillary blood glucose above range (>13.9 mmol/L) during the trial period;
6. The percentage of time of sensor glucose measurement/capillary blood glucose below range (3.0-3.8 mmol/L) during the trial period;
7. The percentage of time of sensor glucose measurement/capillary blood glucose below range (<3.0 mmol/L) during the trial period;
8. The mean sensor glucose concentration;
9. The glucose management indicator (GMI);
10. The glycemic variability (%CV);
11. The risk of incidence of hypoglycemia events;
12. To risk of incidence of hyperglycemic events.

Chapter 4
Safety and Adverse Events

4. Safety and Adverse Events

4.1 Definition of Adverse Events

The included hospitalized T2D patients with insulin therapy might develop hypoglycemia, hyperglycemia events with/without diabetic ketoacidosis or with/without hyperosmotic state, or other adverse events during the trial period.

4.1.1 Hypoglycemia

Hypoglycemia is defined as mild, moderate, and severe: mild hypoglycemia: blood glucose level <3.9 mmol/L; moderate hypoglycemia: blood glucose level <3.5 mmol/L; severe hypoglycemia: blood glucose level <2.8 mmol/L.

Severe hypoglycemia events are defined as a capillary glucose level of less than 2.2 mmol/L or an episode that requirement of assistance of another person due to severe impairment in consciousness or behavior.

4.1.2 Acute hyperglycemia complications of type 2 diabetes

Capillary blood glucose >20.0 mmol/L, and/or ketoacidosis, and/or hyperosmotic status;

4.1.3 Other Adverse Events

An adverse event can be any unfavourable and unintended sign (e.g. an abnormal laboratory finding), symptom (for example nausea, chest pain), or disease temporally associated with the use of a medical product, whether or not considered related to the medical product.

According to the definition by the Food and Drug Administration, USA, severe adverse events are defined as any of the following:

- Life-threatening experience
- Death
- Prolongation of existing hospitalization
- A persistent or significant disability/incapacity
- A congenital anomaly/birth defect

4.2 Recording and Reporting of Adverse Events

All non-serious and serious adverse events will be collected from the time of

signature of informed consent until the end of trial and recorded in the case report form. The occurrence time, clinical manifestation, treatment and duration, outcome should be recorded in detail in the case report form. If an abnormal laboratory examination occurs, the patient should be followed up until the results of the examination return to normal, or to the level before treatment, or to determine if it is not related to the treatment.

If severe adverse events occur in participants, physicians should immediately provide the appropriate care to ensure their safety. Additionally, physicians will report the adverse events and treatment to the Principal Investigator and the Committee within 24 hours and complete the report form.

4.3 Safety Monitoring

In the course of the study, an expert committee will be set up, which consists of two chief physicians who are independent of the two groups. The expert committee will review the trial program and adverse events and make a decision of continuing or suspension of the study. If the patient developed severe hyperglycemia events (capillary blood glucose >20.0mmol/L), or moderate and severe hypoglycemia (capillary blood glucose <3.5mmol/L), the expert committee will review the regimen provided by the RL-DITR. If the recommendation of RL-DITR system is consistent with that of the expert committee, patients continue to be treated in accordance with the insulin dosage titration recommended by the RL-DITR; else if the expert committee does not endorse the insulin dosage titration recommended by the RL-DITR, then the patient will be judged to withdraw from the study and receive treatment by physicians. If patients suffer from severe events such as ketoacidosis, and/or hypertonic coma, the patients will be judged to terminate the study and receive an intravenous infusion of insulin treatment. And if patients suffer from hypoglycemia coma, the patients will be judged to terminate the study and receive an intravenous infusion of glucose therapy.

All case report forms for each participant should be filled out by study staff in a timely manner. The case report form should be double-checked for potential errors or missing data prior to patients leaving the clinic. Original documents and case report forms will be stored in the study office. All data will be double-entered by researcher

staff. Two sets of databases will be generated and tested for consistency using the SAS program. Whenever inconsistencies are found, the data will be corrected by re-examination of the original case report forms or laboratory reports.

Chapter 5
Participant Termination

5. Participant Termination

Patients may be terminated in the following situations: 1) Patients request to withdraw from the study; 2) The expert committee suggests to terminate the study for a medical perspective; 3) Adverse events or other unexpected reasons.

At any time, patients are free to discontinue or withdraw from the study, without prejudice for further treatment. A patient that decides to withdraw from the study will always be asked about the reason(s) and the presence of any adverse events. If possible, they will be seen and assessed by an investigator(s). Adverse events will be followed up.

Chapter 6
Statistical Analyses

6. Statistical Analyses

6.1 Sample Size Estimate

The design of this study is testing for paired means to explore the effect of RL-DITR on glycaemic control. The sample size calculation was based on the primary outcome. According to the existing literature[16] and previous diabetic inpatients statuses in Zhongshan Hospital, the mean daily capillary blood glucose was estimated to be 11 mmol/L at baseline, and to be reduced by the standard difference of 2.5 mmol/L after treatment (13,14,16) With a power of 90% and 1-sided α=.025, 13 participants were required. Considering 20% loss of visit and rejection, at least 16 cases were ultimately needed. PASS11 software was used to calculate the sample size.

To be eligible for analysis using CGM, at least 70% of the CGM measurements had to be available per day. Furthermore, at least four eligible days of CGM measurements had to be available per patient. CGM profiles were analyzed based on the recommendations for standardizing the analysis and presentation of glucose monitoring data (15).

6.2 Data Analysis and Interpretation

Values are presented as mean (SD) or median (IQR) or number (%), unless stated otherwise. All statistical analyses are performed with the use of SAS 9.3 software and two side P value less than 0.05 was considered statistically significant. All analyses are performed by intention-to-treat, and all patients are included in the primary analysis and all secondary analyses unless otherwise noted. The change from baseline measurements to the end of the study was analyzed by two-sided paired t-test and a Wilcoxon signed-rank test for the continuous measurements. The difference of the seven-point blood glucose profiles between the first 24 hour and the last 24 hour were analyzed using generalized linear mixed model. And the percentage of time of sensor glucose concentration in TIR is compared between the first 24 hour and the last 24 hour were analyzed using generalized linear mixed model. Missing data are not imputed. The number of events related to capillary glucose concentrations of less than 2.8 mmol/L and more than 20.0 mmol/L is calculated and compared by Fisher's exact

test. Compliance was also assessed according to participants' daily records and the number of returned dosages. Compliance was calculated as the percentage of the number of insulin orders where dosages were actually ordered according to RL-DITR recommendation against the total number of insulin orders.

Chapter 7
Ethical Requirement

7. Ethical Requirement

This study is approved by the Ethics Committee of the Zhongshan Hospital of Fudan University. The study complies with the declaration of Helsinki and Chinese laws and regulations on clinical trials. The study will respect the rights of participants, and written informed consent will be obtained. The study has been registered in ClinicalTrial.gov (NCT05409391).

Chapter 8
Study Confidentiality
8. Study Confidentiality

The results of the study may be published in a medical journal, and we ensure the patient's personal information will not be leaked according to the relevant legal requirements.

Chapter 9

Training of Research Staff

9. Training of Research Staff

Before the first patient is entered into the study, a principle investigator will review and discuss the requirements of the Clinical Study Protocol and related documents with the investigational staff and also train them in any study specific procedures. The Principal Investigator will ensure that appropriate training relevant to the study is given to all of these staff, and that any new information relevant to the performance of this study is forwarded to the staff involved. The Principal Investigator will maintain a record of all individuals involved in the study (medical, nursing and other staff).

10. Reference

1. Cho, N.H., *et al.* IDF Diabetes Atlas: Global estimates of diabetes prevalence for 2017 and projections for 2045. *Diabetes Res Clin Pract* **138**, 271-281 (2018).
2. Stratton, I.M., *et al.* Association of glycaemia with macrovascular and microvascular complications of type 2 diabetes (UKPDS 35): prospective observational study. *Bmj* **321**, 405-412 (2000).
3. Holman, R.R., Paul, S.K., Bethel, M.A., Matthews, D.R. & Neil, H.A. 10-year follow-up of intensive glucose control in type 2 diabetes. *N Engl J Med* **359**, 1577-1589 (2008).
4. Nawaz, M.S., *et al.* Evaluation of current trends and recent development in insulin therapy for management of diabetes mellitus. *Diabetes Metab Syndr* **11 Suppl 2**, S833-s839 (2017).
5. Bashan, E., Herman, W.H. & Hodish, I. Are glucose readings sufficient to adjust insulin dosage? *Diabetes Technol Ther* **13**, 85-92 (2011).
6. Akirov, A., Grossman, A., Shochat, T. & Shimon, I. Mortality Among Hospitalized Patients With Hypoglycemia: Insulin Related and Noninsulin Related. *J Clin Endocrinol Metab* **102**, 416-424 (2017).
7. Davidson, M.B. How our current medical care system fails people with diabetes: lack of timely, appropriate clinical decisions. *Diabetes Care* **32**, 370-372 (2009).
8. Bi, W.L., *et al.* Artificial intelligence in cancer imaging: Clinical challenges and applications. *CA Cancer J Clin* **69**, 127-157 (2019).
9. Esteva, A., *et al.* Dermatologist-level classification of skin cancer with deep neural networks. *Nature* **542**, 115-118 (2017).
10. Kermany, D.S., *et al.* Identifying Medical Diagnoses and Treatable Diseases by Image-Based Deep Learning. *Cell* **172**, 1122-1131.e1129 (2018).

11. Rigla, M., García-Sáez, G., Pons, B. & Hernando, M.E. Artificial Intelligence Methodologies and Their Application to Diabetes. *J Diabetes Sci Technol* **12**, 303-310 (2018).
12. Liang, H., *et al.* Evaluation and accurate diagnoses of pediatric diseases using artificial intelligence. *Nat Med* **25**, 433-438 (2019).
13. Umpierrez, G.E., *et al.* Randomized study comparing a Basal-bolus with a basal plus correction insulin regimen for the hospital management of medical and surgical patients with type 2 diabetes: basal plus trial. *Diabetes Care* **36**, 2169-2174 (2013).
14. Umpierrez, G.E., *et al.* Comparison of inpatient insulin regimens with detemir plus aspart versus neutral protamine hagedorn plus regular in medical patients with type 2 diabetes. *J Clin Endocrinol Metab* **94**, 564-569 (2009).
15. 6. Glycemic Targets: Standards of Medical Care in Diabetes-2019. *Diabetes Care* **42**, S61-s70 (2019).
16. Umpierrez, G.E., *et al.* Randomized study of basal-bolus insulin therapy in the inpatient management of patients with type 2 diabetes (RABBIT 2 trial). *Diabetes Care* **30**, 2181-2186 (2007).

Supplementary Information. Questionnaires

Table of Contents

1. Recommended insulin dosage (retrospective simulation study of the internal cohort) 24

2. Specialist evaluation (retrospective simulation study of the internal cohort) 26

3. Specialist evaluation (retrospective simulation study of the external cohort) 28

4. Physicians evaluation (prospective study) 30

5. Satisfaction survey form (proof-of-concept trial) 31

Questionnaire 1. Recommended insulin dosage (retrospective simulation study of the internal cohort)

For each case, please give your recommended insulin dosages.

1. Reviewer ID *

____

2. Case ID*

____

3. Your clinical experience (years) *

____

4. Insulin regimens*

○basal

○premixed

○basal bolus

5. Basal insulin dosage① (u) *

____

Dependent on 1st option in question 4

6. Prebreakfast premixed insulin dosage① (u) *

____

Dependent on 2nd option in question 4

7. Predinner premixed insulin dosage② (u) *

____

Dependent on 2nd option in question 4

8. Prebreakfast bolus insulin dosage① (u) *

____

Dependent on 3rd option in question 4

9. Prelunch bolus insulin dosage② (u *

____

Dependent on 3rd option in question 4

10. Predinner bolus insulin dosage③ (u) *

____

Dependent on 3rd option in question 4

11. Basal insulin dosage④ (u) *

____

Dependent on 3rd option in question 4

12. Comments(option)

____

Questionnaire 2. Specialist evaluation (retrospective simulation study of the internal cohort)

For each case, please answer the following questions.

1. Reviewer ID*

____

2. Case ID*

____

3. **Plan Acceptability**: Would the regimen be acceptable in clinical settings? (Multiple selection) *

	Acceptable	Unacceptable
Plan 1	○	○
Plan 2	○	○
Plan 3	○	○
Plan 4	○	○

4. **Plan Effectiveness**: How effective do you think the insulin regimen brings glucose within the normal range? *

1-->5 for very poor control --> very good control

	very poor control (1)	relatively poor control (2)	average (3)	relatively good control (4)	very good control (5)
Plan 1	○	○	○	○	○
Plan 2	○	○	○	○	○
Plan 3	○	○	○	○	○
Plan 4	○	○	○	○	○

5. **Plan Safety**: How much risk do you think the insulin regimen leads to hypoglycemia? *

1-->5 for very high risk --> very low risk

	very high risk	relatively	fair risk	relatively low	very low risk

	(1)	high risk (2)	(3)	risk (4)	(5)
Plan 1	○	○	○	○	○
Plan 2	○	○	○	○	○
Plan 3	○	○	○	○	○
Plan 4	○	○	○	○	○

6. Comments(option)

____

Questionnaire 3. Specialist evaluation (retrospective simulation study of the external cohort)

For each case, please answer the following questions.

1. **Reviewer ID*** ________________________________

2. **Case ID*** __________________________________

3. **Plan Acceptability**: Would the regimen be acceptable in clinical settings? (Multiple selection) *

	Acceptable	Unacceptable
Plan 1	○	○
Plan 2	○	○

4. **Plan Effectiveness**: How effective do you think the insulin regimen brings glucose within the normal range? *

1-->5 for very poor control --> very good control

	very poor control (1)	relatively poor control (2)	average (3)	relatively good control (4)	very good control (5)
Plan 1	○	○	○	○	○
Plan 2	○	○	○	○	○

5. **Plan Safety**: How much risk do you think the insulin regimen leads to hypoglycemia? *

1-->5 for very high risk --> very low risk

	very high risk (1)	relatively high risk (2)	fair risk (3)	relatively low risk (4)	very low risk (5)
Plan 1	○	○	○	○	○
Plan 2	○	○	○	○	○

6. **Preferred Plan**: Which insulin regimen do you think is better? *

○Plan 1 ○Plan 2

7. **Comments(option) ________________________________**

Questionnaire 4. Physicians evaluation (prospective study)
For each case, please answer the following questions.

1. Reviewer ID *

____

2. Date *

____

3. Case ID *

____

4. AI Plan Acceptable: Would the AI regimen be acceptable in clinical settings? *

○Acceptable

○Unacceptable

5. AI Plan Effectiveness: How effective do you think the AI regimen brings glucose within the normal range? *

Very poor control				Very good control
①	②	③	④	⑤

6. AI Plan Safety: How much risk do you think the AI regimen leads to hypoglycemia? *

Very high risk				Very low risk
①	②	③	④	⑤

7. Would you select the AI plan to treat the patient? *

○YES

○NO

Questionnaire 5. Satisfaction survey form (proof-of-concept trial)

If you have used this AI system in a clinical scenario, please make your selection based on the questions and options below.

Your ID Number*

____

Please select the item that best fits your situation:

1-->5 for very dissatisfied/disagree --> very satisfied/agree *

	1	2	3	4	5
I have become proficient in using the AI system.	○	○	○	○	○
The AI system saved my time.	○	○	○	○	○
I think the AI system has a simple and easy to understand interface.	○	○	○	○	○
I often encountered problems when using the AI system.	○	○	○	○	○
I believe the AI system provides better	○	○	○	○	○

control of patients' blood glucose levels.					
I believe the AI system is safe.	○	○	○	○	○
I think it is easy to regulate blood glucose using the AI system.	○	○	○	○	○
I think the recommendatio ns of the AI system are clear and easy to understand.	○	○	○	○	○
I feel more confident when using the AI system.	○	○	○	○	○
I believe most users can learn to use the AI system very quickly.	○	○	○	○	○
I would like to use the AI system in my future practice.	○	○	○	○	○

I would recommend the AI system to other doctors.	○	○	○	○	○
My overall satisfaction with the AI system.	○	○	○	○	○

What do you think are the problems that still exist with insulin-assisted decision-making systems?

____

Supplementary Figure 1. Outpatient simulation study of our RL-based model.

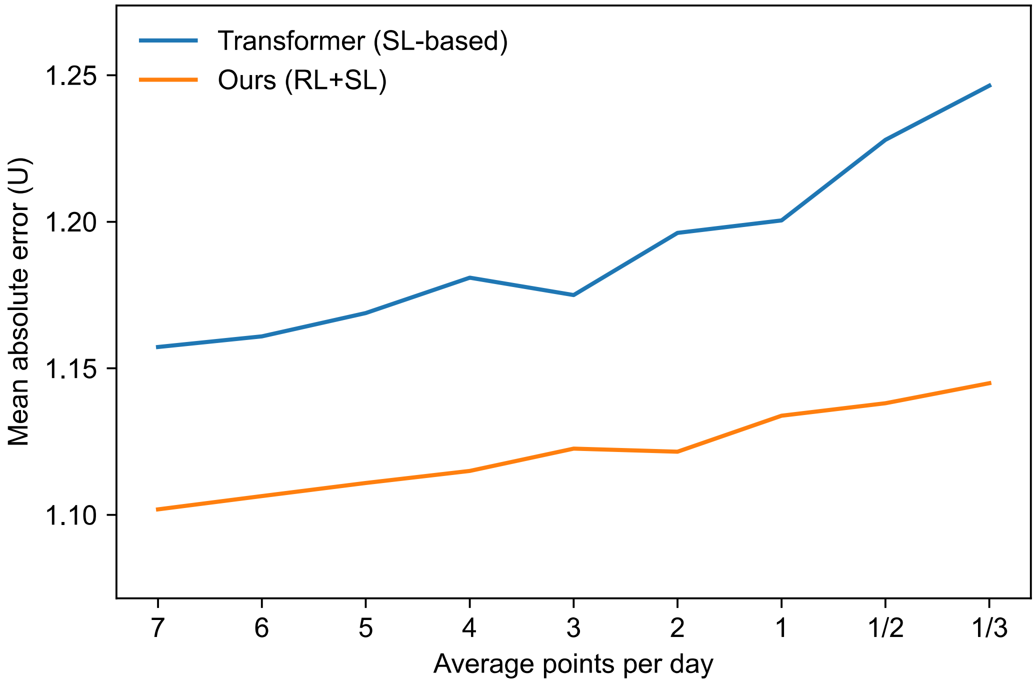

The plot demonstrates the generalizability of our AI system in simulated outpatient settings with various data scenarios, outperforming the supervised learning model Transformer ($P<0.001$, one-sided t-test). The simulation was conducted on the internal test set. The x-axis represents the average number of glucose data points measured per day. The y-axis represents the mean absolute error (MAE), where lower values indicate better performance. The shaded regions depict standard deviation.

Supplementary Table 1. Performance evaluation of our RL-based model in outpatient test set.

	MAE	WIS score
MLP	3.928±0.460	-0.004±0.261
CNN	1.844±0.370	0.120±0.411
LSTM	3.136±0.399	-0.853±0.601
Transformer	1.310±0.191	0.163±0.412
Ours	1.094±0.203	0.315±0.517

Performance evaluation of our RL-based model versus other SL-based methods in an external outpatient test set (n=27). Measured by mean absolute error (MAE) and weighted importance sampling (WIS) score with standard deviation. MLP: multi-layer perception; CNN: convolutional neural network; LSTM: long-short term memory networks.

Supplementary Table 2. List of input features

Feature types	Feature names
**Demographic information and physical examination**	Age
	Gender
	Height
	Weight
	Body mass index
	Heart rate
	Respiration rate
	Systolic blood pressure
	Diastolic blood pressure
**Medical history**	Disease diagnosis
**Symptom**	Thirst
	Polydipsia
	Polyuria
	Emaciation
	Weight loss
	Blurred vision
	Chest pain
	Chills
	Coma
	Cough
	Diarrhea
	Dizziness
	Dyspnea
	Excessive hunger
	Fatigue
	Fever
	Foamy urine
	Frequent micturition
	Headache
	Hyperhidrosis
	Impaired vision
	Involuntary hand movements
	Nausea
	Nocturia
	Numbness in both lower extremities
	Numbness of limbs
	Palpitations
	Polyphagia
	Poor sleep
	Vomiting

**Laboratory testing**	Total carbon dioxide
	Calcium
	Calcium phosphate product
	Potassium
	Chloride
	Magnesium
	Sodium
	Nitrite
	Anion gap
	Albumin
	Albumin/globulion (ratio)
	Alanine aminotransferase
	Alkaline phosphatase
	Lactate dehydrogenase
	Aspartate aminotransferase
	Direct bilirubin
	Total bilirubin
	Total bile acid
	Free triiodothyronine(FT3)
	Free thyroxine(FT4)
	Thyroid stimulating hormone(TSH)
	Total triiodothyronine(TT3)
	Total thyroxine(TT4)
	Adrenocorticotropic hormone(ACTH)
	Cortisol
	Urine PH
	White blood cell count
	Red blood cell count
	Red blood cell microscopy
	Yeast
	Crystallization inspection
	Urine calcium/urine creatinine
	Urine albumin
	Urine albumin-to-creatinine ratio
	Urine bilirubin
	Urobilinogen
	Urine protein
	Urine calcium
	Urine creatinine
	Urine potassium
	Urine output
	Urine phosphorus

	Urine chlorine
	Urine magnesium
	Urine sodium
	Urine glucose
	Urine ketone bodies
	Urine creatinine determination
	Bacteria
	Serum uric acid
	Urea
	Creatinine
	Estimated glomerular filtration rate
	β-Hydroxybutyrate
	Fasting C-peptide
	Fasting insulin
	Glycated albumin
	Glycated hemoglobin
	Ketone bodies
	Serum PH
	Leukocyte
	Total white blood cells
	Monocytes
	Monocyte percentage
	Lymphocytes
	Lymphocyte percentage
	Neutrophils
	Neutrophil percentage
	Basophils
	Basophil percentage
	Eosinophils
	Eosinophil percentage
	Platelets
	Mean platelet volume
	Platelet volume
	Large platelet ratio
	Red blood cells
	Hematocrit
	Mean corpuscular volume
	Mean hemoglobin concentration
	Hemoglobin
	LDL cholesterol
	Triglycerides
	HDL cholesterol

	Free fatty acid
	Total cholesterol
	Jaundice index
	Hemolytic index
**Capillary glucose measurements**	Capillary blood glucose
**Prescriptions**	Total enery (kcal/d)
	Carbohydrate
	Fat
	Protein
	Insulin dosage
	Insulin categories
	Anti-hyperglycemic medications dosage
	Anti-hyperglycemic medications categories

Supplementary Table 3. Consort-AI checklist

Section	Item	CONSORT 2010 Item[a]		CONSORT-AI Item	Addressed on Page No[b]
Title and Abstract					
**Title and Abstract**	1a	Identification as a randomised trial in the title	CONSORT-AI 1a,b Elaboration	(i) Indicate that the intervention involves artificial intelligence/machine learning in the title and/or abstract and specify the type of model.	Page1-2
	1b	Structured summary of trial design, methods, results, and conclusions (for specific guidance see CONSORT for abstracts)		(ii) State the intended use of the AI intervention within the trial in the title and/or abstract.	Page1-2
Introduction					
**Background and objectives**	2a	Scientific background and explanation of rationale	CONSORT-AI 2a (i) Extension	Explain the intended use of the AI intervention in the context of the clinical pathway, including its purpose and its intended users (e.g. healthcare professionals, patients, public).	Page3-5
	2b	Specific objectives or hypotheses			Page4-5
Methods					
**Trial design**	3a	Description of trial design (such as parallel, factorial) including allocation ratio			Page27
	3b	Important changes to methods after trial commencement (such as eligibility criteria), with reasons			NA
**Participants**	4a	Eligibility criteria for participants	CONSORT-AI	State the inclusion and exclusion criteria at the level of participants.	Page27

			4a (i) Elaboration		
			CONSORT-AI 4a (ii) Extension	State the inclusion and exclusion criteria at the level of the input data.	Supplementary Note1
	4b	Settings and locations where the data were collected	CONSORT-AI 4b Extension	Describe how the AI intervention was integrated into the trial setting, including any onsite or offsite requirements.	Page27
**Interventions**	5	The interventions for each group with sufficient details to allow replication, including how and when they were actually administered	CONSORT-AI 5 (i) Extension	State which version of the AI algorithm was used.	Page16
			CONSORT-AI 5 (ii) Extension	Describe how the input data were acquired and selected for the AI intervention.	Page28, Supplementary Note2
			CONSORT-AI 5 (iii) Extension	Describe how poor quality or unavailable input data were assessed and handled.	Supplementary Note2
			CONSORT-AI 5 (iv) Extension.	Specify whether there was human-AI interaction in the handling of the input data, and what level of expertise was required of users.	Page27, Supplementary Note2
			CONSORT-AI 5 (v) Extension	Specify the output of the AI intervention	Page27, Extended Figure 5a
			CONSORT-AI 5 (vi) Extension	Explain how the AI intervention's outputs contributed to decision-making or other elements of clinical practice.	Page27

**Outcomes**	6a	Completely defined pre-specified primary and secondary outcome measures, including how and when they were assessed			Page28-29, Supplementary Note 1
	6b	Any changes to trial outcomes after the trial commenced, with reasons			NA
**Sample size**	7a	How sample size was determined			Page29, Supplementary Note 1
	7b	When applicable, explanation of any interim analyses and stopping guidelines			NA
Randomisation					
**Sequence generation**	8a	Method used to generate the random allocation sequence			NA
	8b	Type of randomisation; details of any restriction (such as blocking and block size)			NA
**Allocation concealment mechanism**	9	Mechanism used to implement the random allocation sequence (such as sequentially numbered containers), describing any steps taken to conceal the sequence until interventions were assigned			NA
**Implementation**	10	Who generated the random allocation sequence, who enrolled participants, and who assigned participants to interventions			NA
**Blinding**	11a	If done, who was blinded after assignment to interventions			Page27

		(for example, participants, care providers, those assessing outcomes) and how			
	11b	If relevant, description of the similarity of interventions			NA
**Statistical methods**	12a	Statistical methods used to compare groups for primary and secondary outcomes			Page29
	12b	Methods for additional analyses, such as subgroup analyses and adjusted analyses			NA
Results					
**Participant flow** (a diagram is strongly recommended)	13a	For each group, the numbers of participants who were randomly assigned, received intended treatment, and were analysed for the primary outcome			Page11-12
	13b	For each group, losses and exclusions after randomisation, together with reasons			Page11-12
**Recruitment**	14a	Dates defining the periods of recruitment and follow-up			Page 27, Supplementary Note 1
	14b	Why the trial ended or was stopped			NA
**Baseline data**	15	A table showing baseline demographic and clinical characteristics for each group			Figure 6a

**Numbers analysed**	16	For each group, number of participants (denominator) included in each analysis and whether the analysis was by original assigned groups			Page11-12
**Outcomes and estimation**	17a	For each primary and secondary outcome, results for each group, and the estimated effect size and its precision (such as 95% confidence interval)			Page11-12
	17b	For binary outcomes, presentation of both absolute and relative effect sizes is recommended			NA
**Ancillary analyses**	18	Results of any other analyses performed, including subgroup analyses and adjusted analyses, distinguishing pre-specified from exploratory			NA
**Harms**	19	All important harms or unintended effects in each group (for specific guidance see CONSORT for harms)	CONSORT-AI 19 Extension	Describe results of any analysis of performance errors and how errors were identified, where applicable. If no such analysis was planned or done, explain why not.	Page11-12
Discussion					
**Limitations**	20	Trial limitations, addressing sources of potential bias, imprecision, and, if relevant, multiplicity of analyses			Page15
**Generalisability**	21	Generalisability (external validity, applicability) of the trial findings			Page14-15
**Interpretation**	22	Interpretation consistent with results, balancing benefits and harms, and considering other relevant evidence			Page14-16
Other Information					

**Registration**	23	Registration number and name of trial registry			Page16
**Protocol**	24	Where the full trial protocol can be accessed, if available			Supplementary Note 1
**Funding**	25	Sources of funding and other support (such as supply of drugs), role of funders	CONSORT-AI 25 Extension.	State whether and how the AI intervention and/or its code can be accessed, including any restrictions to access or re-use.	Page30

[a] We strongly recommend reading this statement in conjunction with the CONSORT 2010 Explanation and Elaboration for important clarifications on all the items.

[b] Indicates page numbers to be completed by authors during protocol development.
