# Supplementary material for: Optimized glycemic control of type 2 diabetes with reinforcement learning: a proof-of-concept trial
Source: Nat Med. 2023 Sep 14;29(10):2633–42. doi: 10.1038/s41591-023-02552-9 (PMC10579102; doi:10.1038/s41591-023-02552-9)
Supplement: Supplementary file 2 — Reporting Summary [file 41591_2023_2552_MOESM2_ESM.pdf]

nature portfolio

Corresponding author(s): Guangyu Wang

Last updated by author(s): Aug 3, 2023

Reporting Summary

Nature Portfolio wishes to improve the reproducibility of the work that we publish. This form provides structure for consistency and transparency in reporting. For further information on Nature Portfolio policies, see our Editorial Policies and the Editorial Policy Checklist.

Statistics

For all statistical analyses, confirm that the following items are present in the figure legend, table legend, main text, or Methods section.

n/a	Confirmed	
☐	☒	The exact sample size (*n*) for each experimental group/condition, given as a discrete number and unit of measurement
☐	☒	A statement on whether measurements were taken from distinct samples or whether the same sample was measured repeatedly
☐	☒	The statistical test(s) used AND whether they are one- or two-sided<br>*Only common tests should be described solely by name; describe more complex techniques in the Methods section.*
☒	☐	A description of all covariates tested
☒	☐	A description of any assumptions or corrections, such as tests of normality and adjustment for multiple comparisons
☐	☒	A full description of the statistical parameters including central tendency (e.g. means) or other basic estimates (e.g. regression coefficient) AND variation (e.g. standard deviation) or associated estimates of uncertainty (e.g. confidence intervals)
☐	☒	For null hypothesis testing, the test statistic (e.g. *F*, *t*, *r*) with confidence intervals, effect sizes, degrees of freedom and *P* value noted<br>*Give P values as exact values whenever suitable.*
☒	☐	For Bayesian analysis, information on the choice of priors and Markov chain Monte Carlo settings
☐	☒	For hierarchical and complex designs, identification of the appropriate level for tests and full reporting of outcomes
☐	☒	Estimates of effect sizes (e.g. Cohen's *d*, Pearson's *r*), indicating how they were calculated

Our web collection on statistics for biologists contains articles on many of the points above.

Software and code

Policy information about availability of computer code

Data collection	No special software or code was used to collect the data. Data preparation was done using Pandas (2.0.3) and Python (3.8).
Data analysis	The artificial intelligence models were implemented using Python (3.8) and PyTorch (1.9.0). Clinical studies were analyzed using SAS 9.3. The natural language processing (NLP) pipeline was developed based on ClinicalBERT, publicly available at https://huggingface.co/medicalai/ClinicalBERT. The codes are available for scientific research and non-commercial use on GitHub at https://github.com/rlditr23/RL-DITR.

For manuscripts utilizing custom algorithms or software that are central to the research but not yet described in published literature, software must be made available to editors and reviewers. We strongly encourage code deposition in a community repository (e.g. GitHub). See the Nature Portfolio guidelines for submitting code & software for further information.

Data

Policy information about availability of data

All manuscripts must include a data availability statement. This statement should provide the following information, where applicable:

- Accession codes, unique identifiers, or web links for publicly available datasets
- A description of any restrictions on data availability
- For clinical datasets or third party data, please ensure that the statement adheres to our policy

Institutional review board (IRB) approval was obtained from institutions for EHR data collection. Individual-level patient records can be accessible with IRB consent and are not publicly available. De-identified data can be requested by writing to the corresponding authors. All data access requests will be reviewed and (if

successful) granted by the Data Access Committee. Data can be shared only for non-commercial academic purposes and will require a formal material transfer agreement. Individual-level data of clinical trial (Clinicaltrials.gov, NCT05409391) reported in this study are not publicly shared. Upon reasonable request and subject to review, de-identified data may be shared upon approval of an analysis proposal and a signed data access agreement. Generally, all such requests for access to the EHR data or clinical trial data will be responded to within 1 month. The LOINC database is publicly available at https://loinc.org/downloads/.

Human research participants

Policy information about studies involving human research participants and Sex and Gender in Research.

Reporting on sex and gender	The sex distributions of the datasets used in this study have been reported. We do not report results stratified by sex.
Population characteristics	Type 2 diabetes patients between 18 and 75 years of age were recruited for the trial. Their mean HbA1c was 8.8±1.1% at baseline and mean diabetes duration was 12.0±8.9 years.
Recruitment	Participants were recruited from hospitalized type 2 diabetes patients. Inclusion criteria comprised age 18-75 years with type 2 diabetes. Exclusion criteria were pregnancy or breast-feeding, severe edema or peripheral vessel disorders, surgery scheduled during hospitalization. The data collected from inpatients and predominantly Han Chinese, might not be representative for the generalized population, potentially introducing biases.
Ethics oversight	All studies were approved by the Ethical Committee of Zhongshan Hospital, Fudan University, and conducted according to the Helsinki Declaration.

Note that full information on the approval of the study protocol must also be provided in the manuscript.

Field-specific reporting

Please select the one below that is the best fit for your research. If you are not sure, read the appropriate sections before making your selection.

☒ Life sciences ☐ Behavioural & social sciences ☐ Ecological, evolutionary & environmental sciences

For a reference copy of the document with all sections, see nature.com/documents/nr-reporting-summary-flat.pdf

Life sciences study design

All studies must disclose on these points even when the disclosure is negative.

Sample size	A total of 12,981 T2D patients with 119,941 treatment days were included in the model analysis, where the sample size was determined by the data availability. For the clinical trial involving 16 participants, the sample size was determined based on the primary outcome with a power of 90% and one-sided alpha=0.025.
Data exclusions	Exclusions were based on data completeness according to the relevant criteria established in the paper for each experiment.
Replication	Replication was not relevant. We used independent validation cohorts.
Randomization	Samples were randomly allocated to the training, tuning and testing sets.
Blinding	During the data processing, all data was first de-identified to remove any patient related information.

Reporting for specific materials, systems and methods

We require information from authors about some types of materials, experimental systems and methods used in many studies. Here, indicate whether each material, system or method listed is relevant to your study. If you are not sure if a list item applies to your research, read the appropriate section before selecting a response.

Materials & experimental systems

n/a	Involved in the study	
☒	☐	Antibodies
☒	☐	Eukaryotic cell lines
☒	☐	Palaeontology and archaeology
☒	☐	Animals and other organisms
☐	☒	Clinical data
☒	☐	Dual use research of concern

Methods

n/a	Involved in the study	
☒	☐	ChIP-seq
☒	☐	Flow cytometry
☒	☐	MRI-based neuroimaging

Clinical data

Policy information about clinical studies

All manuscripts should comply with the ICMJE guidelines for publication of clinical research and a completed CONSORT checklist must be included with all submissions.

Clinical trial registration	The study is registered with ClinicalTrials.gov NCT05409391.
Study protocol	We have provided the full trial protocol in the manuscript.
Data collection	Participants were enrolled from June 2022 to October 2022. Demographics and medical history, body weight and height, glycated haemoglobin (HbA1c), and insulin regimens and dosages were recorded at enrollment. Capillary glucose concentrations and insulin dosage during intervention were collected using case report form. Continuous glucose monitoring (CGM) (Abbott Freestyle Libre, USA) was also used for each patient to collect continuous glucose values.
Outcomes	The primary outcome was difference in glycemic control as measured by mean daily blood glucose concentration (total, preprandial, post-prandial capillary blood glucose). The secondary endpoints included glucose concentration in the target range (TIR) of 3.9-10.0 mmol/L, glucose concentration above range (10.1-13.9 mmol/L or >13.9 mmol/L) or below range (3.0-3.8 mmol/L or <3.0 mmol/L), and glycemic variability. The difference from baseline measurements to the end of the trial was analyzed by two-sided paired t-test and a Wilcoxon signed-rank test. The difference of mean daily blood glucose was assessed for achieving the pre-specified endpoint.
